# Portable NIR spectroscopy: the route to green analytical chemistry

**DOI:** 10.3389/fchem.2023.1214825

**Published:** 2023-09-25

**Authors:** G. Gullifa, L. Barone, E. Papa, A. Giuffrida, S. Materazzi, R. Risoluti

**Affiliations:** ^1^ Department of Chemistry, “Sapienza” Università di Roma, Rome, Italy; ^2^ Department of Chemical Sciences, University of Catania, Catania, Italy

**Keywords:** NIR, portable NIR, handheld NIR, near infrared spectroscopy, chemometrics

## Abstract

There is a growing interest for cost-effective and nondestructive analytical techniques in both research and application fields. The growing approach by near-infrared spectroscopy (NIRs) pushes to develop handheld devices devoted to be easily applied for *in situ* determinations. Consequently, portable NIR spectrometers actually result definitively recognized as powerful instruments, able to perform nondestructive, online, or *in situ* analyses, and useful tools characterized by increasingly smaller size, lower cost, higher robustness, easy-to-use by operator, portable and with ergonomic profile. Chemometrics play a fundamental role to obtain useful and meaningful results from NIR spectra. In this review, portable NIRs applications, published in the period 2019–2022, have been selected to indicate starting references. These publications have been chosen among the many examples of the most recent applications to demonstrate the potential of this analytical approach which, not having the need for extraction processes or any other pre-treatment of the sample under examination, can be considered the “true green analytical chemistry” which allows the analysis where the sample to be characterized is located. In the case of industrial processes or plant or animal samples, it is even possible to follow the variation or evolution of fundamental parameters over time. Publications of specific applications in this field continuously appear in the literature, often in unfamiliar journal or in dedicated special issues. This review aims to give starting references, sometimes not easy to be found.

## 1 Introduction

In the past 20 years, the interest to the potential of near-infrared spectroscopy (NIRs) pushed the development of portable devices devoted to new applications, since several advantages make the technique a powerful analytical tool:

- The spectroscopic approach is a non-destructive characterization, which allows to have immediate analytical answers that can be correlated to other following characterizations on the same identical sample or its representative portion
- NIR spectroscopy coupled to chemometrics allows targeted or untargeted analysis since the multivariate statistical evaluation of the results allows to develop prediction models
- Results can be reported both as qualitative and quantitative output by means of simple plots that are easily readable even by non-experts in chemometrics or by numerical quantification of the single analyte
- The possibility to perform *in-situ* and online analysis favors industrial applications since spectroscopy can be easily automated and connected to an IoT (Internet of Things) system for direct management of process output
- Increasingly smaller size, lower cost, higher robustness, easy-to-use by operator, portable and with ergonomic profile make NIRs a useful tool for routine characterization.

Chemometrics play a fundamental role to obtain useful and meaningful results from NIR spectra, since prediction models allow to obtain qualitative and quantitative analytical results.

As a consequence, publications of specific applications in this field continuously appear in the literature, often in unfamiliar journal or in dedicated special issues. To have an idea of the scientific interest, inserting “NIR applications” in a search engine like Scopus or Web of Science, more than 13,000 results are listed limiting the search to the last 5 years.

Consequently, to help readers in the search for updated useful starting references, sometimes difficult to locate, in this review have been selected NIRs applications published in the period 2019–2022.

## 2 Portable NIR spectrometers

Miniaturized and portable NIR spectrometers have enhanced the applications of near infrared technology, nowadays evermore frequently applied in on-site and in-field analyses. Originally used by military, handheld NIR was suddenly discovered by industry for on-site fast quality check. Law enforcement, environmental analyses and food quality control fully recognize the potential of portable spectrometers.

Fast and reliable classification and characterizations for safety or authenticity can be easily performed by trained users, not necessarily scientists (Sorak et al., 2012; Rodionova et al., 2016; Crocombe, 2018; Pomerantsev and Rodionova, 2021). The main goal when the miniaturization is carried out for an analytical instrument is to verify that performance is not affected. Portable NIR actually ensure comparable analytical results to laboratory benchtop devices, in both qualitative and quantitative aspects. Weight is a fundamental parameter for portable instruments: compared to 1 kg of Raman and MIR spectrometers, NIR handheld weight is about 100 g and the close future is the integration into cellular phones (Reinig et al., 2018).

NIR spectroscopy is mainly based on overtone bands and combination vibrations of C-H, O-H, N-H, C=O, and C=C bonds (Workman and Weyer, 2012; Siesler et al., 2016). Although the signal occurs about ten to one hundred times lower as intensity with respect to medium infrared, the chemometric prediction models make handheld NIR an useful qualitative and quantitative characterization approach in analyses.

Basically, a portable NIR device is very similar to a laboratory benchtop instrument. Miniaturization involves Micro-Electro-Mechanical or Micro-Opto-Electro-Mechanical optical systems. Two main detectors are usually found: array or single detector. Indium gallium arsenide (InGaAs) is the most used material for detectors because of the lower price.

The following main solutions are actually recognized:

(a) Linear-variable filter instruments (array detector).
(b) MEMS-based FT-NIR instruments (opto-electro-mechanical).
(c) Micro-mirror device (DMD™) (wavelength selector).
(d) Fabry-Perot tunable wavelength filter.
(e) NIR grating.

In Table 1 several examples are reported.

**TABLE 1:** Examples of portable NIR spectrometers available in the market and their monochromator/detector principles.

TrinamiX GmbH, Ludwigshafen, Germany	Linear variable filter instruments
VIAVI Solutions Inc., Santa Rosa, CA, United States	
Texas Instruments, Dallas, TX, United States	Digital micro-mirror device spectrometers
Innospectra Corp., Xinzhu, Taiwan, China	
Spectral Engines, Helsinki, Finland	Fabry Perot tunable filter instrument
Si-Ware Systems, Cairo, Egypt	MEMS FT-NIR spectrometers
Hamamatsu Photonics, Hamamatsu city, Japan	
Southnest Technology, Hefei, Anhui, China	
Insion GmbH, Obersulm,	grating microspectrometers
Germany; OrO Photonics, Xinzhu, Taiwan, China	
Senorics GmbH, Dresden, Germany	NIR scanner with 16 solar cell detectors

Recent publications critically report detector differences (Bec et al., 2021), showing that the signal-to-noise ratio of modern instruments compensates narrower NIR wavelength range.

The sample measurement is highly simplified and makes very easy-to-use the portable NIR spectrometers: by direct contact (but also placing at short distance) it is possible to record characteristic spectra in diffuse reflection.

**FIGURE 1:**
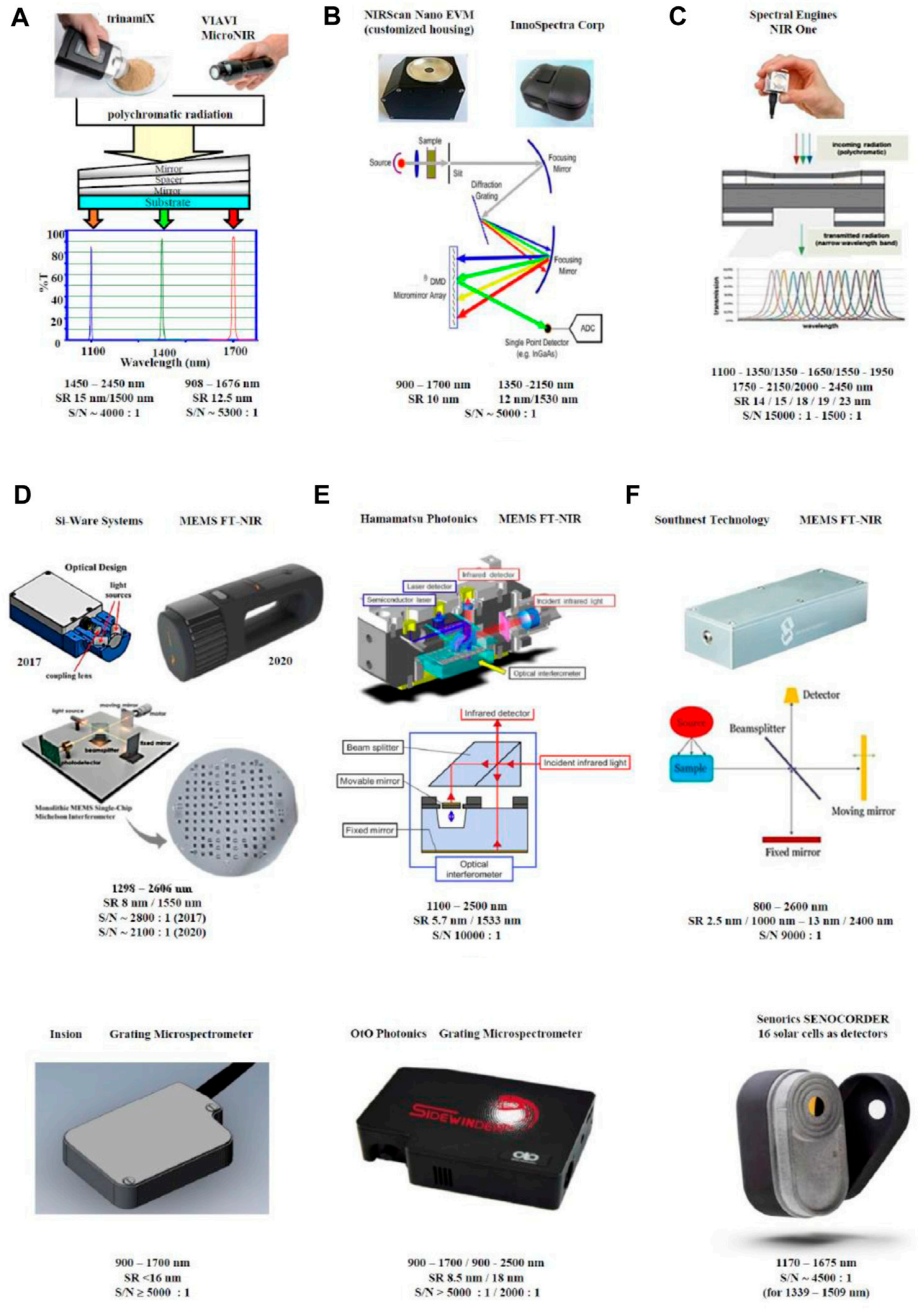
Handheld NIR spectrometers with different monochromator/detector principles and their performance parameters: **(A)** linear variable filter instruments (trinamiX GmbH, Ludwigshafen, Germany; VIAVI Solutions Inc., Santa Rosa, CA, United States); **(B)** digital micro-mirror device (DMD™) spectrometers (Texas Instruments, Dallas, TX, United States; Innospectra Corp., Xinzhu, Taiwan, China); **(C)** Fabry Perot tunable filter instrument (Spectral Engines, Helsinki, Finland); **(D)** MEMS FT-NIR spectrometers (Si-Ware Systems, Cairo, Egypt; Hamamatsu Photonics, Hamamatsu city, Japan; Southnest Technology, Hefei, Anhui, China); **(E)** grating microspectrometers (Insion GmbH, Obersulm, Germany; OrO Photonics, Xinzhu, Taiwan, China) **(F)** NIR scanner with 16 solar cell detectors (Senorics GmbH, Dresden, Germany). Reproduced (CC-BY 4.0 license) from (Bec et al., 2021).

Figure 1 collects several different portable NIR solutions, commercially available. Simplified principles and parameters are briefly described.

### 2.1 Instrument improvements

To improve the stability of handheld near infrared spectrometers, a reference correction was proposed by Wang et al. On the basis of the measurement method, it was designed a contrast sampling mechanism to obtain a reference beam from light source. This configuration ensured that reference beam and light source drift in the same trend. An innovative NIR spectrometer for reference correction was consequently developed. According to the experimental results, the stability of the newly designed spectrometer achieved substantial improvement (Wang et al., 2021).

Phosphor-converted light-emitting diodes (pc-LEDs) are employed in handheld spectrosmeters. A high-performance NIR pc-LED was proposed and results demonstrated that the energy transfer can be used for developing new NIR phosphors (Basore et al., 2021). He et al. proposed an innovative pc-LED device, able to scan in a 5-cm-thick chicken breast so to identify characteristic differences in the spectra. The results indicated that biological tissue penetration can be diagnostic (He et al., 2022). To identify affected chicken breasts, de Cavalho et al. proposed handheld NIR spectroscopy. From the experimental results it was shown that myopathies in meat is correlated to the age of slaughter and handheld NIR can be used as a specific tool (de Carvalho et al., 2020).

Soil texture characterization is usually carried on manually, being consequently time-consuming. Vis-NIR handheld spectroscopy is preferred as sensor to predict soil characteristics (Bene et al., 2020; Benedet et al., 2020; Pham et al., 2021; Andrade et al., 2022; Naimi et al., 2022; Peng et al., 2022; Shrestha et al., 2022; Teixeira et al., 2022; Vohland et al., 2022).

Goi et al. evaluated the possibility of a micro NIR instrument to predict quality parameters in beef. Moisture, fat and protein contents were monitored by a modified PLS analysis. Results proved that the portable instrumentation ensure good levels of prediction (Goi et al., 2022). Grass-fed and grain-fed beef were differentiated by Coombs et al. using NIR spectroscopy with the aim to aid retail and consumer confidence (Coombs et al., 2021).

Yu et al. (2022) prepared innovative OPDs with broad bandwidth, improved responsivity and frequency.

For decades, NIRs demonstrated its potential in industrial process monitoring and quality insurance. Ou et al. (2022). tested a portable NIR in the 850–1700 nm wavelength range. The results from his study demonstrated that the spectrometer, although limited in NIR spectral band detection, can be used in several different sensing applications. Paiva et al. (2022) described the application of diffuse reflectance to liquid and gas samples, enhancing that does not require adaptations. Biodiesel and vegetable oil in diesel blends were determined by comparing benchtop and portable FT-NIR spectrometers.

Bertinetto et al. (2022) investigated the influence of unexpert operator actions in the interpretation of spectra of pig feed.

Miniaturization made possible to develop handheld instruments, so lowering the price of point-of-use analysis. Baumann et al. developed a cheap, handheld DLP-Nano-NIRscan associated to a smartphone for the storage of data. A specific app was developed according to a software design standard. Baumann and coworkers demonstrated the usefulness for plant tissues to determine total nitrogen content (Baumann et al., 2020).

Wang and Liu published a review where they provided a brief summary for the factors that need to be considered in the design of broadband NIR phosphors. Authors expected this review to offer useful guidelines for the development of advanced NIR pc-LEDs (Wang and Liu, 2022).

As a consequence of handheld NIR development, dedicated apps become fundamental tools. Ren and Jia used samples of four drugs as validation objects to test the data classification model, that showed satisficing accuracy and the possibility to overcome internal storage limitations and operating speed (Ren and Jia, 2023).

(Zhou et al., 2022a) used a handheld NIR instrument for powdered sample characterization. The device showed high potentiality in powder analysis and comparative characterization.

Among easy-to-use and cheap analytical instruments, optical multisensor luminescence devices were proposed by Surkova et al. (2022) to determine fat content and adulteration in milk samples.

Applications in the Internet of Things (IoT) have been developed. Classic analytical methods, like mass spectrometry and chromatography, are not compatibles with IoT. NIR devices, having no moving parts, are ideal for IoT applications. Applications in industrial and agricultural field have been reported (Wang et al., 2021).

A critical review collected NIR applications, published between January 2019 and July 2020, focused on advantages of this handheld, cheap and robust analytical technique with non-destructive properties. These characteristics insure fast quality control on food products, raw materials, and ingredients (Bwambok et al., 2020). Applications in quality analysis and authentication of nutraceuticals using NIRs was also published in a review by Nagy et al. (Nagy et al., 2022). Reviewed innovation in handheld NIR and summarized and compared their characteristics (Zhu et al., 2022).

## 3 Applications to food quality characterization and adulterations

Food quality (and consequently food fraud) is often related to profit: the higher is the quality, the higher can be the prize in the market. Frauds or adulterations can result in illicit but important money gain. However, sometimes food fraud can impact the health of customers and consumers. Fraud detection is a challenge to help consumers and to punish food fraudsters.

On the other hand, quality is often a complicated mix of factors not easy to specifically determine or to quantify. NIR spectroscopy creates a specific profile that is the sum of all the specific parameters and can be a fundamental analytical tool. The possibility of managing a “profile” instead of individual analytes allows the NIRs/chemometrics approach to provide an untargeted result that takes into account all the parameters that characterize the sample under examination and guarantees, in the case of food, to characterize a “profile” of quality as a reference even in case of fraud investigation.

Combining NIR spectroscopy and machine intelligence is a nondestructive tool for powdery food evaluation. Zhou et al. presented a handheld device (“NIR-spoon”) for simultaneous evaluation of multi-mixture powdery food. Each mixed powdered sample was analyzed by a “NIR-Spoon” and software was consequently dedicated to the “NIR-Spoon” that resulted in a good accuracy with the possibility of a mobile app (Zhou et al., 2022b). An OPLS-DA model, based on the data from the handheld NIR, showed 84.85% and 86.96% correct classification (Srinuttrakul et al., 2021).

The determination of green pea and peanut adulterations in pistachio by handheld FT-MIR and FT-NIR spectroscopy was proposed by Aykas and Menevseoglu. Pistachio adulteration derives from its high value. A fast FT-NIR methodology to check adulteration was proposed and provided advantages over FT-MIR, being more precise (Aykas and Menevseoglu, 2021). Cashew nut adulteration with Brazilian nut, pecan nut, macadamia nut and peanut was proposed, based on the development of a one-class SIMCA model (Rovira et al., 2023). Zhu et al. evaluated quality traits of oats to support breeding selection (Zhu et al., 2022).

Prediction of meat quality in fresh cut was reported by (An et al., 2022), while after treatment in the abattoir was reported by (Savoia et al., 2020; Savoia et al., 2021). Li et al. proposed a rapid and nondestructive simultaneous characterization of different types of sheep meat cut (Li et al., 2021).

Extra virgin olive oil is among the most known quality made in Italy product. Violino et al. (2022) evaluated the performance of a handheld VIS–NIR system to determine 203 oil samples quality. Ciaccheri et al. reported a bluetooth-connected pocket NIR spectrometer for the analysis of olive oils of different qualities (Ciaccheri et al., 2022) and a similar study was published by Santos and coworkers (Borghi et al., 2020; Santos et al., 2020; Santos et al., 2022).

Escuredo et al. (2021a). proposed a simple portable spectroscopic approach to predict the properties in honeys by multivariate data processing.

Maraphum et al. used a portable NIR spectrometer to determine starch and dry matter amount in fresh cassava tubers. Results suggested that the method can be useful when monitoring the quality of cassava tubers “*in field*” by breeders, with save of time and costs (Maraphum et al., 2020; Maraphum et al., 2022). In coriander oil can be found about the 70% of petroselinic acid, with anti-inflammatory and anti-aging properties. The authenticity of coriander oil and modifications by means of commercial oils was tested by handheld NIR and PLS regression models (Kaufmann et al., 2022). Edible oils are usually adulterated with sesame oil to save production costs. Portable FT-NIR, FT-MIR, and Raman spectrometers were proposed as rapid, simple, and non-invasive method to detect adulteration (Menevseoglu, 2021).

Truffles are well known and very expensive edible mushroom. Black truffles can be however found at lower prices. It is not easy to distinguish truffles without experience and often frauds occur. Kappacher et al. analyzed truffle samples of different species by 4 NIR devices: of these, 3 were miniaturized devices (Risoluti et al., 2020a). The resulting published paper compared the predicting performances of portable vs. benchtop NIR instruments, suggesting a fast, non-destructive and cheap tool (Kappacher et al., 2022).

The raspberry characterization is a procedure laborious and expensive, due to several chemical tests to use. By a NIRs approach, the prediction of raspberry quality was proposed by Gales and coworkers. Anthocyanins and solid soluble concentrations in whole fresh raspberries were easily predicted (Gales et al., 2021).

Sripaurya et al. (2021) designed and implemented a 6-digital-channel portable NIR instrument to determine the quality and the maturity of Gros Michel bananas. Portable NIR to in-field determine “Valencia” orange fruit maturity and avocado was reported by Ncama and coworkers (Ncama et al., 2020a; Ncama et al., 2020b). The evaluation of portable spectrometers performance to characterize table grape and peach quality attributes non-invasively, was proposed by (Donis-González et al., 2020), by (Rouxinol et al., 2022), by (Zhang et al., 2021), by (Ferrara et al., 2022) and by (Beltrame et al., 2021).

Almonds, easy to adulterate to have higher profits because of the lower production costs, were characterized by a developed method for identification of almond flour adulteration by comparing three different portable NIR instruments and SIMCA as classification method (Netto et al., 2023). Bitter almonds contamination in sweet almonds was determined by by the potential of portable NIR (Torres et al., 2021). Wang et al. realized a handheld Vis/NIR prototype to discriminate different origin apples (Wang et al., 2022).

Manuelian et al. evaluated cheese total nitrogen, soluble nitrogen, ripening index, major minerals, and fatty acids by a pocket-size NIR spectrometer. When comparing the accuracy with a benchtop instrument, however, no substantial difference in accuracy resulted. The NIR miniaturization can consequently be an opportunity in dairy industry (Manuelian et al., 2022). Bittante et al. published an invited review with focus on VIS and NIR spectroscopies to predict the cheese chemical composition (Bittante et al., 2022).

Omega-3 supplements are sold at elevated prices and their quality has to be assessed. Hespanhol et al. evaluated the performance of a cheap NIR assisted by a chemometric model to determine the concentration of omega-3 in supplements. This simple, fast and inexpensive approach determined the quality of the supplements and to identify frauds or non-conformities of the market (Hespanhol et al., 2020).

Durum wheat semolina is the raw material for pasta production and, when cooking, are fundamental the protein content to obtain the gluten strength. Cecchini et al. compared two technologies (both non-destructive and rapid), i.e. a low-cost sensor and a handheld NIR spectrometer, to determine semolina quality. Experimental evidence enhanced the possibilities of a small and low cost sensor, easy to use in contact with the sample more than by a laboratory instruments (Cecchini et al., 2021).

Intrinsic and extrinsic properties of rice were studied to give the consumer a rice quality identification. Spectra were collected by a handheld NIR to develop a predictive model (Rizwana and Hazarika, 2020). Jiang and coworkers reported a fatty acid surveillance during the storage of the rice by a handheld near infrared system (Jiang et al., 2020).

Insects are well known as possible human food. Riu and coworkers studied the possibility to classify insect powder by handheld NIR spectrometer. Experimental evidences confirmed this instrumental approach to be a trusted tool of analysis, able to predict macronutrients (Riu et al., 2022).

The moisture content deeply influences the dehydration process to prevent microbial growth and to preserve nutrients and quality of fruits and vegetables. The results of a study proposed by Malvandi showed how a handheld NIR is able to monitor the moisture content (Malvandi et al., 2022a). Pesticide residues determination was presented by Ngo et al. on leafy vegetables using a handheld VIS/NIR instrument. Vegetable samples, like lettuce, oriental mustard, and bok choy, were analyzed. Results showed that pesticide residues on leafy vegetables can be easily detected (Ngo et al., 2022). Recently, handheld and smartphone-based NIR devices have been widely used in agri-food industries as food-scanners, based on their predictive potential and compared with destructive approaches (Goisser et al., 2021). Integrated soluble solid and nitrate content assessment of spinach plants using portable NIRS sensors along the supply chain was also reported by (Torres et al., 2020)

Mishra and Woltering used an innovative approach that was tested to predict moisture and total soluble solids in pear and kiwi fruit (Mishra et al., 2021; Mishra and Woltering, 2021).

Anyidoho et al. (2022). used NIR/Chemometrics for rapid determination of fermentation duration, fermentation index and moisture content of cocoa beans.

The watermelons maturity was determined by two handheld new generation NIR spectrometers were compared in determining soluble solid content, a parameter that proves full watermelon maturity. The results obtained by different pre-processing methods showed the possibility to predict watermelon maturity (Vega-Castellote et al., 2022). Qi et al. discriminated the different varieties of red Jujube (Risoluti et al., 2019a; Qi et al., 2022).

Lycopene ia a red colored carotenoid in tomatoes that shows health benefits in scavenging free radicals. Instead of traditional in laboratory extraction process followed by HPLC analysis, Goisser et al. compared Vis/NIR spectra obtained by three portable Vis/NIR instruments defined food-scanners (Goisser et al., 2020a; Goisser et al., 2020b). The commercialization of fresh tomatoes is based on the soluble solid content as quality parameter. A handheld Vis-NIR spectrometer in interactance geometry discriminated low from high values soluble contents (Brito et al., 2021). Food waste has to be reduced as much as possible. Emsley et al. assessed the NIR potentiality to prove tomato stability during storage. Resulting informations indicated the possibility to predict tomatoes time-after-harvest (Emsley et al., 2022). Arruda de Brito et al. also proposed the possibility to determine important parameters (color, dry matter, etc) by NIR spectroscopy (Arruda de Brito et al., 2022). Handheld NIR for in-field fresh tomato quality control analysis was reported by (Borba et al., 2021) and by (de Oliveira Aguiar et al., 2022).

Gatti and coworkers investigated by handheld NIR milk chocolate, white chocolate and other different percent cocoa chocolates subjected to high temperatures. Good sensibility, specificity, and accuracy values were determined to establish thermal treatments (Gatti et al., 2021).

To assess fishery products authenticity it is fundamental an instrumentation able to give rapid, eco-friendly, cost-effective and easy answer. Varrà et al. verified the possibility to determine the geographic origin of two octopus species by a handeld-ultra-compact NIR. The results suggested the possibility to in-site or on-line monitoring fishery products (Moon et al., 2020; Varrà et al., 2022). Non-destructive characterization of salmon and tuna freshness was proposed by (Cui and Cui, 2021). Fish identification is not easy neither for consumers nor for inspectors, especially when prepared as fillets. To develop fast detectors, Cavallini et al. distinguished very similar fish species by comparing handheld SCiO (Consumer Physics), MicroNIR (VIAVI) with benchtop MPA (Bruker). (Risoluti et al., 2015; Pennisi et al., 2021; Cavallini et al., 2022). Handheld NIR spectroscopy was proposed as discrimination tool between wild and farmed sea bass by (Esposito et al., 2022). Yakes et al. (2021) reported the analysis of two fish species and compared the performance of handheld NIR devices. Currò et al. (2021) proposed handheld NIR instrument as a fast and green method to check the origin of cuttlefish. Yu et al. (2020) assessed by rapid and nondestructive application of handeld NIRs the freshness of tilapia fillets.

By means of a calibrated image analysis, (Tugnolo et al., 2021) and by (Grassi et al., 2021) evaluated the maturation of olives. Comparative analysis was reported by optical fiber probe/benchtop NIR device. Considering the vis/NIR model good performance, an algorithm was applied.

Butteroil has sensory characteristics, economic importance, and because of the high value it can be replaed by other cheaper fats. The potential of handheld NIR spectroscopy to detect butteroil adulterations was suggested to help producers and inspectors in the supply chain (da Silva Medeiros et al., 2023).

Pepper spice adulteration is a growing problem. Evidences from analyses by a handheld NIR demonstrated that adulteration of white pepper can be easily identified (Chen et al., 2022). Oliveira and coworkers proposed PLS-DA and PLSR models (based on handheld NIR spectra) as adulteration identifiers of Paprika powder (Oliveira et al., 2020).

Ouyang and coworkers proved the possibility to predict by handheld NIR spectroscopy the total nitrogen content in pork meat (Kucha and Ngadi, 2020) or frozen pork meat (Ouyang et al., 2020). Portable NIR and machine learning was proposed by (van Kollenburg et al., 2020) and by Parastar and coworkers (Parastar et al., 2020) to determine the authenticity of chicken fillet. Beef, pork, and chicken quantification in ground meat was reported by (Silva et al., 2020).

Milk quality can be evaluated by the content of fats, proteins, lactose and total solids. Adulteration of goat milk by cow milk was proposed by (dos Santos et al., 2021) and by Muniz and Cuevas-Valdés (Muñiz et al., 2020). To authenticate milk geographical origin, Zhang et al. proposed the use of portable NIR spectroscopy to correctly and rapidly detect the adulteration (Zhang T. et al., 2022). To rapidly and conveniently detect the compositions of milk, a handheld detector was developed by Yang et al. 120 raw samples helped to set up the PLSR model to predict the main composition (Yang et al., 2020). A review on the recent advances in NIR portable spectrometers and the applications to milk, cheese and dairy powders was published by (Pu et al., 2021). That is widely supplied in the market has three main active ingredients, including. Ashie and coworkers determined, by portable NIR spectroscopy, active ingredients like silybin, silychristin and silydianin, and isosilybin in milk thistle extract because of their valuable functions for the human body. Results showed that SNV and first derivative discriminate silybin and isosilybin (Ashie et al., 2021). Water adulteration in raw milk of bovine was detected by Ehsani et al. (Ehsani et al., 2022) and by (Riu et al., 2020) who applied classification and regression techniques to spectra from handheld near infrared spectroscopy for facile and rapid detection. Risoluti et al. (Scala et al., 2018) and (Galvan et al., 2022) demonstrated that low-cost handheld NIRs and EDXRF spectrometry coupled to chemometrics can differentiate authentic cow and goat milks from whey mixed ones.

Human milk banks recently expanded because of the newborns growing need. However, human milk composition analyses are far from routine possibility. Jorge dos Santos and coworkers developed a comparative spectroscopic approach by a portable MicroNIR to compare composition of human milk (Jorge dos Santos et al., 2021) and its conservation (dos Santos et al., 2022). Melendreras et al. (2022) characterized breast milk composition along the lactation period. The results proved the richness of near infrared spectra, and specific bands provided excellent quantitative models.

Lanza et al. (2021) compared benchtop with handheld NIR characterization to discriminate refrigeration periods of chicken breast. PLS-DA indicated correct shelf-life prediction.

A fast online estimation of quail eggs freshness was proposed by (Brasil et al., 2022) and by (Cruz-Tirado et al., 2021).

Alcohol content is essential for the quality control of beers. To this aim, a new rapid and direct multivariate method was proposed and validated using a portable NIR spectrometer and PLS regression, supported by a parallel GC-FID method (da Costa Fulgêncio et al., 2022). Foam parameters are related to beer quality and depend on the protein amount. Viejo et al. used machine learning to predict 54 proteins. Fifteen parameters with handheld NIR assessed significant correlations to physical parameters (Viejo et al., 2020).

Escuredo et al. (2021b) determined quality parameters for potatoes by a MicroNIR device, such as dry matter and reducing sugars. PCA and modified PLS models demonstrated to be useful for potato processors. NaSO_3_ acts as browning inhibitor when storing potato slices. However, sulfur dioxide residues can be formed, very dangerous to health. NIR hyperspectral imaging was demonstrated to be able to classify SO_2_ residues (Bai et al., 2020).

Technicians and producers of dairy farm forage need rapid and reliable equipment to control the quality, better if in unexpensive and easy-to-use way. Rego and coworkers presented a portable-NIR-based procedure to analyze the nutritional values of dairy farm forage. By means of Internet of Things (IoT) tools, spectra can be sent to cloud to be processed and accessible to any device. A chemometric model was developed and validated (Rego et al., 2020).

Productivity and wellness of animals can be improved by precision nutritional composition forage. Near infrared spectroscopy demonstrated to be a very useful tool to determine the nutritional content of forage (Rukundo et al., 2021a; Rukundo et al., 2021b).

Beć et al. (2022) published a thematic review to collect the applications of food-analysis miniaturized NIRs, enhancing challenges and perspectives.

## 4 Applications to drugs

The surveillance of medicines is of fundamental importance to guarantee and protect the consumer, especially when the medicines are life-saving or have a strong impact on the patient’s therapy. Consequently, the surveillance of medicine devices is fundamental. NIR spectroscopy plays an extremely interesting role, given the possibility of checking the product without necessarily taking sample aliquots. The ability to carry out analyses, for example, directly inside the packaging blister is now known. Several authors have published interesting scientific applications to drug surveillance and characterization.

Caillet et al. tested six devices’ utility and usability in detecting substandard and falsified medicines: four handheld spectrometers (two near infrared and two Raman), one portable MIR spectrometer and a single-use paper analytical device (Caillet et al., 2021; Greco et al., 2023a; Greco et al., 2023b). Laboratory evaluation of twelve portable devices for medicine quality screening was proposed by Zambrzycki and coworkers, including portable instruments (Zambrzycki et al., 2021).

Illicit-drug seizures are a growing problem since the forensic drug identification is not easy to perform, especially in case of new psychoactive substances. Kranenburg and coworkers (Kranenburg et al., 2022a; Kranenburg et al., 2022b; Kranenburg et al., 2022c) and Awotunde and coworkers (Awotunde et al., 2022) introduced a handheld NIR with a 1,300–2,600 nm range. A specific chemometric model was developed for forensic samples. Authors report that it only requires few reference spectra for training the model, thus eliminating the need of extensive training sets including mixtures. The early detection of emerging street drugs by near infrared spectroscopy and chemometrics was extensively studied by Risoluti and coworkers (Risoluti et al., 2016; Materazzi et al., 2017a; Risoluti et al., 2019b; Risoluti et al., 2019d; Risoluti et al., 2020f).

An innovative screening platform was realized and validated for the detection of cannabinoids on-site in hemp seed oil, aimed to food safety control in market products. This completely automated tool is based on a miniaturized NIR in wireless mode that allows rapid and accurate sample processing and the early detection of residual cannabinoids in oils, including CBD, Δ9-THC and the Δ9-THCA (Risoluti et al., 2019c; Risoluti et al., 2019d; Risoluti et al., 2020b; Risoluti et al., 2020c; Risoluti et al., 2020d) and amphetamine (Risoluti et al., 2020e).

A discrimination of falsified Amoxicillin capsules using heterogeneous NIR spectroscopic devices for training and testing of a support vector machine (Hattori et al., 2021). A handheld NIR spectrometer was employed to quantify heroin was described by Hattori et al. samples by PLS regression and applied to determin heroin casework samples in the concentration range 4.49%–88.05% (w/w). The approach showed good results as repeatability and precision and the results demonstrated an acceptable model performance as on-site heroin detection instrument (Chen et al., 2021).

When considering a pharmaceutical manufacturing process, the ensurance of quality is needed either in the final product or in intermediates. A Raman probe and a portable NIR were applied at the blending process exit. Both the predictive models were tested for accuracy, precision, operating range, measurement frequency, placement, reliability, robustness (Panikar et al., 2021). Hattori et al. (2022). improved algorithms to screen falsified or substandard amoxicillin capsules. FT-NIR and portable wavelength dispersive NIR spectrometers validated the proposed approach. Zhong et al. (2022) proposed the handhelded NIR spectroscopy for the evaluation of coating uniformity of digestion-aid tablets.

A specific review was proposed by Usman et al. to resume the different applications of portable or miniaturized medium-IR, near-IR and Raman instruments for efficient control of quality in pharmaceutical products. These portable spectrometers demonstrated to be a powerful tool to identify of counterfeits, adulterated, fraudulent, falsified, and substandard pharmaceutical capsules or drops (Usman et al., 2020).

The possibility to evaluate Low-Cost Optical Spectrometers to detect falsified medicines is a growing need. Medicines are often counterfeit and is fundamental to warrant rapid, sensible and nondestructive methodologies for the characterization of products. Handheld NIR was proposed by (Wang et al., 2020) and by Assi et al. to authenticate branded or generic antibiotics. The resulting NIR spectra showed characteristic spectra for the main component(s) (Assi et al., 2021). Three-dimensional printing is a revolutionary technology in pharmaceuticals, enabling the personalisation of flexible-dose drug products and 3D printed polypills (polyprintlets). Polyprintlets were non-destructively characterized using a handheld NIR and calibration models were proposed by (Trenfield et al., 2020).

There is a market of seized controlled substances in combination with drug-of-abuse in which new substances psychoactive have to be identified by rapid and reliable presumptive drug testing, better if on-site. Spectroscopic techniques show several advantages because spectra are specific and non-invasive analyses are possible. NIRs is a promising technique for forensic drug detection directly “on-scene”. Several portable spectrometers were sold in the past years. Kranenburg et al. realized a dataset of spectra with 430 samples, including illicit-drugs, NPS, adulterants, bulking-agents and excipients. By this dataset, illicit-drugs spectra were usable by institutes that not easily get access to controlled substances and can be of help in developing chemometric models to detect illicit-drugs (Kranenburg et al., 2022d; Kranenburg et al., 2022e).

Since 2019, COVID-19 has exploded as a global pandemy. The possibility to have false vaccines gave illusion of security, highering the exposure to the virus, and increasing the risk of infection. Assi et al. proposed a non-destructive handheld NIR approach for verification of COVID-19 vaccines. Bands corresponding to the mRNA active ingredient were detected by NIR. PCA allowed the validation of COVID-19 vaccines (Assi et al., 2022). As a parallel security procedure, alcohol-based hand sanitizers were recommended as a strategy to minimize contamination. By handheld NIR with PLS-DA, models were realized to check for conformity of commercial hand sanitizing products, while PCA was proposed to test a exploratory study. Results indicated that NIR spectroscopy with chemometrics are a promising tool to discriminate hand sanitizers and able to detect the right ethanol concentrations (Pasquini et al., 2020; Silva et al., 2021).

Aerial parts of Cannabis spp. can be easily purchased; however, in the cannabis not only is present cannabidiol, but Δ9-tetrahydrocannabinol can also be detected and its psychotropic effects are well known and its presence in high concentration is illegal in almost all the European countries. Officers need to test the Δ9-tetrahydrocannabinol concentration in questioned samples. In addition, a portable equipment is of sure interest for farmers to control the THC content in time. Duchateau et al. analyzed hemp flowers and compared results from benchtop or portable NIR devices. A GC-FID analysis determined in parallel the related THC concentration. The proposed models clearly discriminated legal and illegal cannabis samples on the base of European and Swiss laws (Duchateau et al., 2020).

Women use medroxyprogesterone when hormonal therapy is needed. In case of falsification, health risk could result for consumers. A handheld spectrometer with open-sourced software was tested by Eady and coworkers to evaluate vials of Medroxyprogesterone acetate injectable suspensions from different suppliers and results were validated (Ead et al., 2021).

Anti-leishmanial pentamidine bioconjugates based on PLGA-PEG and hyaluronic acid were described by Scala et al. (Ca et al., 2018).

## 5 Applications in medical diagnostic

NIR spectroscopy demonstrated to be a very helpful tool in medical diagnostic. The non-invasive spectral characterizations, with the fundamental help of chemiometric evaluation and machine learning, have been scientifically proved to be a fast and sensitive preliminary approach for a preliminary health check. Several publications can be found in recent literature.

Traumatic brain injury can lead to hematomas or edemas inside the cerebral tissue. To this end, low-cost, portable and easy-to-handle devices can be fundamental in continuous monitoring. NIR based techniques have been proposed as good solution. Vera et al. used time resolved Monte Carlo simulations in a handheld NIR study. Results showed that mean partial pathlengths, photon measurement density functions and time dependent contrasts are influenced by lesions, proving to be robust means for diagnose or monitoring (Vera et al., 2022). For the strict control of solid oral preparations, Zhang et al. (2022) studied how to determine the content of active pharmaceutical ingredient by a portable NIR sensor in the production line.

Hafiz and coworkers proposed a new hand-held camera for retinal imaging including white or NIR-LED adjacent to each other, able to illuminate by NIR without additional beam-splitters or filters. The camera was adapted to be interfaced to Android-based smartphones to determine ocular safety analysis (Hafiz et al., 2022).

Bhattacharya et al. presented the development of a NIR-portable instrument to detect acute ischemia stroke by means of albumin in human blood serum. The test on human blood samples showed good linearity to determine the concentration of albumin in human blood (Bhattacharya et al., 2022). Similar studies related to the detection of hematologic pathologies were reported by Gullifa et al. (Risoluti et al., 2019e; Risoluti et al., 2020f; Risoluti et al., 2020g; Gullifa and Risoluti, 2021). The dynamic control of lymphatic and vascular systems was published by (Wang et al., 2022).

An unusual study was proposed by Ni and coworkers, who evaluated handheld-NIR possibility to *in vivo* analyze healthy tissues and to correlate the results with age. Partial Least Square regression showed that vibrational spectroscopy non-destructive techniques can be used as useful tools to relate interactions between physiology and nutrition (Ni et al., 2022a). Ni et al. also evaluated the ability of an handheld NIR to relate spectral informations to food and energy intake, satiation, and satiety data. Results showed the potentiality of *in-vivo* NIR spectroscopy to differentiate tissues (Ni et al., 2022b). Clemson et al. (2022) proposed a portable and non-contact system for skin surveillance.

Diabetes is a life-threatening disease and needs constant monitoring of blood glucose levels. Yu and coworkers proposed a mobile application for handheld NIR useful to non-professional users. The experimental results showed that the portable and light NIR spectrometer can be easily used (Yu et al., 2021a). A NIR-LEDs-Based detection system was also proposed by Badriah et al. to measure blood sugar levels on diabetic care (Badriah et al., 2022).

Belay et al. (2020) presented a three-segment grating spectrometer, aimed to help in cancer diagnosis. The results experimentally proved that this solution significantly improves the spectrometer signal. With the same view, Bonapace et al. (2021) described how to determine methylglyoxal adducts content by portable handheld NIR. MicroNIR/chemometrics determination of hydroxyurea occupational exposure was also proposed as a new tool in work-surveillance and prevenction (Risoluti and Materazzi, 2018).

In a review published by Huang et al., a smartphone-based NIR fluorescent imaging technology was proposed. Smartphones can improve diagnoses being a tool for real-time diagnosis, even in remote regions (Huang et al., 2021). Hasan et al. proposed a state of the art review, collecting the predictive and non-invasive techniques of hemoglobin level (Hasan et al., 2021).

## 6 Applications to agriculture

Portable systems can be of fundamental usefulness for in-site material characterization and quality analysis in agricultural. Miniaturized NIRs were proposed in material-sensing applications. To validate the performance, several classification algorithms were compared (Behera et al., 2020).

B1 and B2 fumonisin rapid detection in corn was described by Shen and coworkers by smartphone-based handheld NIRs/chemometrics (Shen et al., 2022).

ESI(±)FT-ICR MS and portable microNIR were proposed as a new analytical tool to characterize Robusta coffees. A published study confirmed that both can be efficiently applied to coffee quality control, with many advantages such as speed and analytical reliability (Correia et al., 2020). Baqueta and coworkers directly evaluated cupping profiles in coffee blends via portable NIR and PLS-DA model. Results were presented as industrially interesting and the method could help coffee professionals in cup evaluation (Baqueta et al., 2021). A one-class classification method was proposed to control agroforestry-grown coffees by (Manuel et al., 2022).

Forage analysis by NIR spectroscopy had many advancements since 1970. Due to instrumentation, computers and chemometric algorithms improvements, it is actually the most used approach for the routine analysis by forage producers, plant breeders, animal nutritionists, cattle farmers, and feed companies. Berzaghi et al. (2021) reported a comparison among three different portable instruments and a benchtop lab instrumentation, using many forage samples.

Phenotyping information is fundamental for sugar industry. Gaci and coworkers proposed a micro-, low-cost, portable narrow VIS-NIR instrument to get a PLS predictive model (Gaci et al., 2022). Henrique da Silva Melo and coworkers evaluated portable spectroscopy for the quantification of brix and pol in a sugar production plant (Henrique da Silva Melo et al., 2022).

Edible oils, modified lipids, industrial oils, and biofuels can be produced by Brassica. Analytical methods based on Portable NIRs and NIR-Hyperspectral Imaging were applied to characterize seeds species (da Silva Medeiros et al., 2022). Jiang et al. (2022) and (Bilal et al., 2020) proposed a fast way to determine peanuts acidity index. Yu and coworkers classified the high oleic acid peanuts by comparing portable and benchtop NIRs. The accuracy of distinction was 100% with both the instruments (Yu et al., 2020). Jiang et al. (2021) and (Giussani et al., 2021) used a handheld NIR device to analyze the acid values during the storage of edible oils. Grossi et al. (2020) proposed a procedure to determine solid fat by single-wavelength NIRs.

Yao et al. developed a rapid NIR authentication procedure for exhaust oils used in frying potato chips. A miniaturized NIR sensor showed how handheld vibrational spectrometers can provide a rapid *in-situ* identification of oil type and could be a surveillance tool of the products (Yao et al., 2021).

Data from three different NIRs were analyzed to compare coriander seeds. Three instruments were compared in their prediction ability. 200 authentic coriander seeds spectra were compared with 90 adulterated samples (McVey et al., 2021). Often consumers of seedlings or fruits have troubles to recognize if that product really is what is indicated by the merchant. Consequently, it becomes particularly crucial to evaluate the marketing process. Vinhandelli et al. used PC-LDA or PLS-DA algorithms to test the ability of NIR spectroscopy to discriminate cultivars (Vinhandelli et al., 2023). Nitrogen assessment in strawberries by handheld FT-NIR was also proposed by Wu and coworkers (Wu et al., 2020).

A cost-effective and fast analysis of nutrient content in cotton leaves by handheld NIR spectroscopy was reported by (Prananto et al., 2021).

Quantitative assay of Aflatoxin B1 in maize was proposed by Deng et al. Their NIR system was realized and employed to characterize maize samples with different mildew degrees (Deng et al., 2022). High-quality production of tobacco leaves needs identification of deep green infection. Janqiang et al. proposed an identification methodology for deep green tobacco infections using a portable NIR (Jianqiang et al., 2020).

Detecting of apple valsa cancer is useful to prevent diseases and to check apples yield and quality. Zhao et al. proposed portable NIRs and Raman scattering spectrometers in reflection mode using machine learning to predict infection degrees (Zhao et al., 2021). Qiao and coworkers proposed apple sugar content determination by handheld tool connected to a cellular phone (Qiao et al., 2020). Apple hardness can be determined by non-destructive, as proposed by (Malvandi et al., 2022b) and by (Ma et al., 2021a).

The citrus industry is always searching for instrumental methods able to determine in real time and *in situ* the modifications of fruit development or storage, with a special focus on vitamin C. A NIR-based model was proposed to vuate the ripeness (Santos et al., 2021). Jahani et al. (2020) proposed a miniaturized handheld NIR as a preliminary test of adulteration in lime juices coupled with classification methodologies.

The current practice by potato growers for tissue testing is based on petiole chemical analysis rather than leaf analysis. AbuKmeil and Al-Mallahi estimated nutrients in potato intact fresh leaves by a portable Vis-NIR spectrophotometer and the parallel chemical analyses were done on petioles following the official methods of the AOAC. The Lasso models showed a fair distribution of nitrogen, phosphorus, potassium, calcium, magnesium, and zinc with coefficient of determination values above 0.5 and acceptable to excellent RPD values (abuKmeil and Al-Mallahi, 2022).

Wang and coworkers (Wang et al., 2020; Wang et al., 2020; Sun et al., 2020; Wang et al., 2021; Jin et al., 2021; Ren et al., 2022) developed a handheld tool to check the freshness of tea.

The contamination of bee pollen by pyrrolizidine alkaloids is actually determined by LC-MS. De Jesus Inacio and coworkers suggested the use of fast NIR spectroscopy as alternative and accurate approach (De Jesus Inacio et al., 2020).

In the livestock and poultry breeding industry, the slurry is a mixture of urine, feces, flushing water, and disinfectant. The composition varies greatly when returning to the field, due to several different influencing interferences. Liang et al. developed a NIR method to *in-situ* determine the slurry characteristics (Liang et al., 2022).

## 7 Applications to forensics

The use of portable NIR devices in forensic analysis has recently undergone an important boost as it makes it possible to acquire fundamental information directly at the crime scene, before starting the currently necessary sampling, custody and pre-treatment procedures. In addition, these characterizations are not destructive, nor do they modify the analyzed sample. These two aspects are peculiar in forensic investigations as they make it possible to ensure the objectivity of the experimental evidence even by non-specialized, but simply trained, personnel. Traces of human blood are among the most important evidences in criminal investigations. Blood samples have to be identified and collected immediately without contamination in the crime scene. Fonseca and coworkers evaluated the possibility to unambiguously identify human blood in stains found in floor tiles by handheld NIR spectroscopy. Authors reported that hierarchical models result in a significant scientific improvement useful for the identification of human blood stains at crime scenes (Fonseca et al., 2022).

Criminals often use common household cleaners, that are strong corrosive solutions, such as, in order to stun the victim. NIR handheld spectrometer was demonstrated to determine corrosive solutions through plastic bottles. Morillas and Frascione evaluated corrosive and harmless substances, to test the real NIR performance. Each predictive model identified the corrosive substances, enhancing the ability of this technique to pre-screen corrosive substances (Morillas and Frascione, 2022).

A modular handheld Vis-NIR spectrophotometer collected spectra from ink signatures by different ball-point pens, as a non invasive tool for ink identification in forensics (Ristova et al., 2022). As new approach in forensic chemistry, NIR/Chemometrics was proposed for the characterization of toners when questioned documents examination is requested (Materazzi et al., 2017b; Risoluti et al., 2018a).

It was proposed to solve illicit drug analysis by an ultra–portable NIR linked to a mobile application, demonstrating the possibility to display the result within 5 seconds (Coppey et al., 2020).

Determination of explosives was reported by Risoluti et al. on human hands using handheld MicroNIR (Risoluti et al., 2018b) and by Santonocito et al. using an optical array (Santonocito et al., 2022).

## 8 Applications to textiles

Textiles are daily used by all the people in the world. Especially in high fashion, quality and price can greatly change and often it is not easy to identify genuine products only by visualization. The possibility to easily characterize textiles allows to warrant consumers. Textiles are characterized by obvious differences in raw materials (wool, cotton, synthetics) but often small differences (blended versus pure filaments or the optimization of the appearance of polyester versus cotton) can result in significant quality assurance issues. NIR spectroscopy has demonstrated considerable potential in component characterization and feature differentiation.

Handheld NIR was proposed as good solution to textile authentication and identification by Yan et al. who demonstrated to be simply the comparison with reference spectra. PCA combined with SIMCA resulted the best discrimination models (Yan and Siesler, 2018).

Silk is a precious textile not only because of its physical and chemical properties, but also for its beauty. Cotton or polyester are the mainly mixed fibers to optimize the best look (Kumagai et al., 2004). Being not easy the distinction of silk blends from pure silk, portable NIR was tested for a fast characterization of dress composition, including a quantitative answer (Liu et al., 2013; Guifang et al., 2015). The silk was also blended with polyester and fully characterized by PLS calibration (Yan et al., 2020).

Identification of ancient textile fibers becomes fundamental for conservation of textile relics. Fiber identification methods require sampling and slicing cultural relics for observation under an optical microscope or a scanning electron microscope. Handheld NIR was applied to the identification of fibers from four Qing Dynasty textile relics, and resulted in a good spectral comparison (Li et al., 2021; Ding et al., 2021).

## 9 Applications to materials

Benchtop and handheld molecular spectrometers have been successfully applied in material identification. Compared to FT-MIR spectra, near infrared profiles allow to complete informations and often solve problems by simply comparing characteristic profiles instead of single band interpretation and assignment.

“To recycle” will be a must for the next generations. Great impulse is actually given to recycle the most common polymers. Handheld NIR was demonstrated to be a very easy-to-use tool to classify them with the aim of a safe recycle (Xiong et al., 2016).

Eder and coworkers described and discussed the possibility to use and transfer analytical techniques, including NIR, for in-field characterization of photovoltaic modules. Polymeric compounds of the photovoltaic modules were analyzed and characterized without modules dismantling, transporting into the lab, cutting and analyzing in conventional bench-top spectrometers (Eder et al., 2020). Catauro et al. characterized bioactive ferrous citrate–silica hybrid materials obtained by a sol–gel synthesis.

## 10 Applications to wood, cellulose, lignin and lignite

Wood species can be identified by Vis-NIR spectroscopy: this approach is based on light absorption scattering due to the wood characteristics. However, innovative methodologies based on diffuse reflectance demonstrated the possibility to classify many different woods and portable NIR instruments can be easily used for in-field characterizations.

A handheld Vis-NIR system was realized and, to simplify the interpretation of the spectra, wood classification was obtained by PCA scores (Ma et al., 2021b). The development of a low-cost handheld spectrometer to detect wood defects was proposed by (Sandak et al., 2020).

Characterizations from a standing tree minimize and optimize the time requested since eliminate the transport, the transformation in powder and the storage. Ramadevi et al. (2022) estimated the yield of Kraft pulp by portable NIR spectrometer. A fast characterization of holocellulose and lignin in wood by Kernel extreme learning machine was proposed by (Yang et al., 2020).

Off-line determination of diverse wood quality aspects by a portable NIR sensor was demonstrated by Sandak et al. in glue-laminated timber. The chemometric model specifically developed was demonstrated to be helpful in prediction of the total delamination and detailed delamination length (Sandak et al., 2021).

Three moisture predicting models in Para rubber timber were proposed by Noypitak et al. The handheld NIR spectrometer coupled to a smartphone facilitated the quantification of real-time moisture content. An android application was specifically settled up to manage the instrument. Unknown samples allowed to validate the rsults from the predictive equation (Noypitak et al., 2022). A similar approach was proposed by Puttipipatkajorn (Puttipipatkajorn and Puttipipatkajorn, 2020).

Lim et al. proposed to determine the densities of cross linking in latex by an innovative method based on portable NIR (Lim et al., 2021).

## 11 Applications to natural extracts

Natural extracts can result in complex mixtures with unknown composition if by-products or contaminations are not monitored and eventually eliminated through subsequent purifications. Consumer protection therefore becomes fundamental both for the bodies in charge of surveillance and for the producer who, in self-protection, controls his own product. Zhuang et al. proposed a quantitative model to monitor active components in Radix Astragali extract. The quantitative model was stressed by comparing a usual FT-NIR with a portable NIR. PLSR models indicated that the calibration model can significantly improve the adaptability to new samples (Zhuang et al., 2022).

The market of Turmeric is increasing since curcuminoids are recognized to give health benefits. Spectroscopic methods with chemometrics are able to quantify turmeric to control food quality and allow analysis speed, versatility, portability and no need of any pretreatments. Khongkaew et al. published a study with 5 calibration models for the quantification of curcuminoids in turmeric both by benchtop and portable instrument. The results indicated benchtop and portable methods in good agreement, confirming the suitability of portable devices in food quality control by analyses *in situ* (Khongkaew et al., 2022).

Menevseoglu proposed portable FT-NIR, FT-MIR, and Raman spectrometers to identify adulterations in black seed oil by. SIMCA and PLSR models were applied to predict the adulterant levels (Menevseoglu, 2022).

## 12 Other applications

### 12.1 Applications to cultural heritage

Cultural heritage is the field with the greatest application interest as the non-invasive and portability characteristics of spectroscopy fully embrace the needs of conservation and respect for works that continue over time to bear witness to the excellence of the past. For example, wall paintings are art works based on ancient technologies and materials typical of civilizations from the past. Imaging instrumentation allows the study of the materials and the characterization of pigments.

Asscher and Halevi presented comparative results by stationary or portable modified digital camera, showing the strong environmental influence on the image (Asscher and Halevi, 2022).

In the project “Leonardesque Artists beyond the Visible”, non-invasive portable imaging and spectroscopic-based approach were proposed to obtain useful informations like the description of pigments composition, binder preparation and painters’ technique identification. Results underlined a specific painting technique in each author (Galli et al., 2021).

### 12.2 Applications to geology

The characterization of mafic and ultramafic rocks is important to determine relationships with specific. Adams et al. demonstrated that the X-ray accurate determination can be completed by handheld VIS and NIR analysis (Adams et al., 2021).

### 12.3 Applications to waste materials

Plastic waste classification is fundamental to set the right recycle Diffuse reflectance spectra of several reference and commercial polymers allowed to give fast, nondestructive, in-site characterizations by applying different pretreatments. The results proved that PCA allows to separate different plastic materials (Yang et al., 2020).

### 12.4 Aquaphotomics

The spectrum of the water in aqueous systems gives information on covalent OH and hydrogen bonds since they are deeply influenced. In water NIR spectra, the information results as a function of internal and external factors. Aquaphotomics evaluates by near infrared spectra the groundwaters, not influenced by other factors like temperature or humidity.

Results published by Kovacs and coworkers demonstrated that groundwater samples give specific fingerprint and unicity of the spectroscopic profile that can be used as an indicator of water changes (Kovacs et al., 2022).

Soil-to-water ratios were calculated by Vis-NIR spectra to predict the electrical conductivity by (Gozukara et al., 2022)

A special issue, dedicated to aquaphotomics, was published in the open-access journal Molecules (Muncan and Tsenkova, 2023).

### 12.5 Applications to fuels

Systematic adulterations of gasoline, ethanol and diesel make fundamental the possibility to have analyitical tools, able to determine fuel integrity. Tosato et al. (2020) characterized 115 seized fuels and proved the efficiency of portable near infrared spectrometers assiste by chemometrics. Diesel quality can be evaluated by the “condensation point”. In-field fast determination of condensation point could minimize the costs. Wan et al. (2021) collected representative spectra using a handheld NIR to setup a prediction model.

Motor and crude oil can be improperly mixed, as found in Brazil by companies of energy sector. Santos and coworkers showed the possibility of handheld NIR spectroscopy to detect these frauds. Discrimination potential resulted in 100% of specificity and precision (Santos et al., 2021).

Environment protection pushes to renewable energies like bioenergy. Fuel surveillance to meet the european rules is consequently needed. Hand-held near spectroscopy was tested on wood chip samples. Results showed a satisfactory reliability because each measure is acquired in seconds and the non-destructive analysis allows to efficiently control fuel quality (Toscano et al., 2022). Pellet recently was studied because of its storage cost and combustion efficiency. The possibility to trace pellet quality is important, since fraud behaviors could impact consumers’ health. Usual pellet analyses are costly and time-consuming Mancini and coworkers defined a handheld-NIRs approach to propose a fast and automatic classification of pellet (Mancini et al., 2020).

### 12.6 Applications to micro- and nano-materials

Orsi et al. setted up and calibrated a handheld NIR method to determine ROS, because of its impact in Photodynamic therapies. The approach is based on the fluorescence emission in the near-infrared spectral range at *λ* = 1,270 nm. Several nanostructures have been proposed as able to produce reactive oxygen species voted to medicine therapies (tumours in deep tissue) (Orsi et al., 2022).

Microplastics residues are a growing environmental problem. A review proposed by Tirkey and Upadhyay collected and highlighted innovative approaches in microplastics sampling and identification, including NIR (Tirkey and Upadhyay, 2021).

### 12.7 Applications to asphalt

Modern asphalts are often the result of modifiers inclusions. These modifiers increase sustainability, lower rutting and low-temperature cracking. To this end, a portable molecular sensing technology was proposed, consisting in a pocket-sized near-infrared molecular sensor, able to detect recycled materials. It was demonstrated that the resulting fingerprint of the bituminous materials helps the classification (Jahangiri et al., 2021). The same authors proposed a smartphone-based approach (Barri et al., 2020).

## 13 Chemometrics

Private companies always more ask for on-site analysis by portable instruments, better if spectroscopic devices. Schoot et al. presented a new prediction model from portable NIR data to predict protein content in pig feed (Schoot et al., 2022).

With increasing availability of handheld NIR spectrometers, is of interest the possibility to transfer calibration model from benchtop to portable NIR. Rukundo and coworkers calibrated a benchtop NIR and transferred calibrations to a benchtop and portable instruments. Model transferring however was not direct and required data treatments (Rukundo et al., 2022).

Advantages in milk analysis by NIR spectrometers was proposed by (Said et al., 2022).

Various preprocessing techniques and their combinations were comparatively proposed by Sarkar et al. to quantify soluble solids in kiwi fruits (Sarkar et al., 2020). The content of dry matter when harvesting was proved to have correlation with soluble solids in Kakadu plum samples (Bobasa et al., 2021). De Freitas and coworkers (de Freitas et al., 2022) and Wokadala and coworkers (Wokadala et al., 2020) determined the fruit dry matter content at harvest in Brazil, while the detection of internal physiological disorders was determined by (Mogollón et al., 2020). Portable optical instruments were specifically studied to determine fruit ripeness (Abasi et al., 2021), even directly on the by McCormick and Biegert (McCormick and Biegert, 2021). Pissar et al. (2021), (Zhang et al., 2022; Zhang et al., 2022), (Guo et al., 2020) and (Fan et al., 2020) described the usefulness of a portable NIR to determine apple quality.

Yang et al. (2022) and (Yu et al., 2021b) determined soluble solids content in pears based on VIS-NIR and model analysis and variable selection. Wu et al. (2021). settled up a non-destructive characterization of pears by a home-made NIR and a chemometric prediction model. Mulisa Bobasa and coworkers evaluated 4 different positions to achieve NIR spectra of Kakadu plum fruits, thus monitoring fruit chemical composition (Mulisa Bobasa et al., 2020). The nonlinear optical properties of monodispersed diphenylpolyynes were also studied by (Fazio et al., 2014).

Xiao and Chen presented an innovative research on NIR data analysis by using Machine Learning algorithms to study *in-vivo* the human skin characteristics. NIR showed sure potential to develop a cheap, handheld and powerful, skin characterization tool (Ca et al., 2018; Scala et al., 2018; Risoluti et al., 2020h; Xiao and Chen, 2022).

## Abbreviations

NIRs, Near Infrared spectroscopy; PCA, Principal Component Analysis; PLSR, Partial least square regression; PLS-DA, Partial Least Squares—Discriminant Analysis; SIMCA, Soft Independent Modelling of Class Analogies.

## References

[B1] Abasi S. Minaei S. Jamshidi B. Fathi D. (2021). Development of an optical smart portable instrument for fruit quality detection. IEEE Trans. Instrum. Meas. 70, 1–9. 10.1109/TIM.2020.3011334 33776080

[B2] abuKmeil R. Al-Mallahi A. (2022). “Detecting nutrients in potato plants based on visible/near infrared in-field spectral measurements,” in Proceedings of the 2022 (ASABE Annual International Meeting).

[B3] Adams C. Dentith M. Fiorentini M. (2021). Characterization of altered mafic and ultramafic rocks using portable xrf geochemistry and portable vis-nir spectrometry. Geochem. Explor. Environ. Anal. 21. 10.1144/geochem2020-065

[B4] An J. Li Y. Zhang C. Zhang D. (2022). Rapid nondestructive prediction of multiple quality attributes for different commercial meat cut types using optical system. Food Sci. Anim. Resour. 42, 655–671. 10.5851/kosfa.2022.e28 35855268 PMC9289799

[B5] Andrade R. Mancini M. Teixeira A. F. D. S. Silva S. H. G. Weindorf D. C. Chakraborty S. (2022). Proximal sensor data fusion and auxiliary information for tropical soil property prediction: Soil texture. Geoderma 422, 115936. 10.1016/j.geoderma.2022.115936

[B6] Anyidoho E. K. Teye E. Agbemafle R. Amuah C. L. Y. Boadu V. G. (2021). Application of portable near infrared spectroscopy for classifying and quantifying cocoa bean quality parameters. J. Food Process. Preserv. 45. 10.1111/jfpp.15445

[B7] Arruda de Brito A. Campos F. dos Reis Nascimento A. Damiani C. Alves da Silva F. de Almeida Teixeira G. H. (2022). Non-destructive determination of color, titratable acidity, and dry matter in intact tomatoes using a portable vis-NIR spectrometer. J. Food Compos. Anal. 107, 104288. 10.1016/j.jfca.2021.104288

[B8] Ashie A. Lei H. Han B. Xiong M. Yan H. (2021). Fast determination of three components in milk thistle extract with a hand-held NIR spectrometer and chemometrics tools. Infrared Phys. Technol. 113, 103629. 10.1016/j.infrared.2021.103629

[B9] Asscher Y. Halevi S. (2022). Characterizing the pigments in wall paintings: Comparing portable and stationary multiband remote sensing imaging systems. Springer Proc. Mater. 16, 71–82. 10.1007/978-3-031-03795-5_14

[B10] Assi S. Arafat B. Abbas I. Evans K. (2022). Evaluation of portable near-infrared spectroscopy for authentication of MRNA based COVID-19 vaccines. PLoS ONE 17, e0267214. 10.1371/journal.pone.0267214 35507562 PMC9067670

[B11] Assi S. Arafat B. Lawson-Wood K. Robertson I. (2021). Authentication of antibiotics using portable near-infrared spectroscopy and multivariate data analysis. Appl. Spectrosc. 75, 434–444. 10.1177/0003702820958081 32830991 PMC8645310

[B12] Awotunde O. Roseboom N. Cai J. Hayes K. Rajane R. Chen R. (2022). Discrimination of substandard and falsified formulations from genuine pharmaceuticals using NIR spectra and machine learning. Anal. Chem. 94, 12586–12594. 10.1021/acs.analchem.2c00998 36067409

[B13] Aykas D. P. Menevseoglu A. (2021). A rapid method to detect green pea and peanut adulteration in pistachio by using portable FT-MIR and FT-NIR spectroscopy combined with chemometrics. Food control. 121, 107670. 10.1016/j.foodcont.2020.107670

[B14] Badriah S. Bahtiar Y. Andang A. (2022). Near infrared LEDs-based non-invasive blood sugar testing for detecting blood sugar levels on diabetic care. J. Biomim. Biomater. Biomed. Eng. 55, 183–191. 10.4028/p-vthp40

[B15] Bai X. Xiao Q. Zhou L. Tang Y. He Y. (2020). Detection of sulfite dioxide residue on the surface of fresh-cut potato slices using near-infrared hyperspectral imaging system and portable near-infrared spectrometer. Molecules 25, 1651. 10.3390/molecules25071651 32260173 PMC7180573

[B16] Baqueta M. R. Coqueiro A. Março P. H. Valderrama P. (2021). Multivariate classification for the direct determination of cup profile in coffee blends via handheld near-infrared spectroscopy. Talanta 222, 121526. 10.1016/j.talanta.2020.121526 33167236

[B17] Barri K. Jahangiri B. Davami O. Buttlar W. G. Alavi A. H. (2020). Smartphone-based molecular sensing for advanced characterization of asphalt concrete materials. Meas. J. Int. Meas. Confed. 151, 107212. 10.1016/j.measurement.2019.107212

[B18] Basore E. T. Wu H. Xiao W. Zheng G. Liu X. Qiu J. (2021). High-power broadband NIR LEDs enabled by highly efficient blue-to-NIR conversion. Adv. Opt. Mater. 9, 1660. 10.1002/adom.202001660

[B19] Baumann L. Librelotto M. Pappis C. Helfer G. A. Santos R. O. dos Santos R. B. (2020). NanoMetrix: An app for chemometric analysis from near infrared spectra. J. Chemom. 34, 3281. 10.1002/cem.3281

[B20] Beć K. B. Grabska J. Huck C. W. (2022). Miniaturized NIR spectroscopy in food analysis and quality control: Promises, challenges, and perspectives. Foods 11, 1465. 10.3390/foods11101465 35627034 PMC9140213

[B21] Bec K. B. Grabska J. Huck C. W. (2021). Principles and applications of miniaturized near-infrared (NIR) spectrometers. Chem. Eur. J. 27, 1514–1532. 10.1002/chem.202002838 32820844 PMC7894516

[B22] Behera A. R. Kumar A. Suresh H. Pratap M. Selvaraja S. K. Pratap R. (2020). An ultra-portable vis-NIR spectrometer with an integrated light source for chemometric applications. J. Electrochem. Soc. 167, 167515. 10.1149/1945-7111/abc7e8

[B23] Belay G. Y. Hoving W. Van Der Put A. Van Erps J. Vervaeke M. Thienpont H. (2020). “Design and prototyping of a multi-segment grating for a broadband and miniaturized spectrometer,” in Proc. Proc. SPIE Int. Soc. Opt. Eng (USA: SPIE).

[B24] Beltrame K. K. Gonçalves T. R. Março P. H. Gomes S. T. M. Matsushita M. Valderrama P. (2021). Pseudo-univariate calibration based on NIR spectroscopy in the determination of anthocyanins and antioxidant activity in grape juices. J. Braz. Chem. Soc. 32, 1131–1136. 10.21577/0103-5053.20210007

[B25] Benedet L. Faria W. M. Silva S. H. G. Mancini M. Guilherme L. R. G. Demattê J. A. M. (2020). Soil subgroup prediction via portable X-ray fluorescence and visible near-infrared spectroscopy. Geoderma 365, 114212. 10.1016/j.geoderma.2020.114212

[B26] Benedet L. Faria W. M. Silva S. H. G. Mancini M. Demattê J. A. M. Guilherme L. R. G. (2020). Soil texture prediction using portable X-ray fluorescence spectrometry and visible near-infrared diffuse reflectance spectroscopy. Geoderma 376, 114553. 10.1016/j.geoderma.2020.114553

[B27] Bertinetto C. G. Schoot M. Dingemans M. Meeuwsen W. Buydens L. M. C. Jansen J. J. (2022). Influence of measurement procedure on the use of a handheld NIR spectrophotometer. Food Res. Int. 161, 111836. 10.1016/j.foodres.2022.111836 36192968

[B28] Berzaghi P. Cherney J. H. Casler M. D. (2021). Prediction performance of portable near infrared reflectance instruments using preprocessed dried, ground forage samples. Comput. Electron. Agric. 182, 106013. 10.1016/j.compag.2021.106013

[B29] Bhattacharya R. Ahirwar D. Biswas B. Bhutani G. Chowdhury S. R. (2022). A NIRS based device for identification of acute ischemic stroke by using a novel organic dye in the human blood serum. Lect. Notes Electr. Eng. 886, 43–53. 10.1007/978-3-030-98886-9_4

[B30] Bilal M. Xiaobo Z. Arslan M. Tahir H. E. Azam M. Junjun Z. (2020). Rapid determination of the chemical compositions of peanut seed (Arachis hypogaea.) using portable near-infrared spectroscopy. Vib. Spectrosc. 110, 103138. 10.1016/j.vibspec.2020.103138

[B31] Bittante G. Patel N. Cecchinato A. Berzaghi P. (2022). Invited review: A comprehensive review of visible and near-infrared spectroscopy for predicting the chemical composition of cheese. J. Dairy Sci. 105, 1817–1836. 10.3168/jds.2021-20640 34998561

[B32] Bobasa E. M. Netzel M. E. Cozzolino D. Phan A. D. T. Sultanbawa Y. (2021). Measurement of total soluble solids and moisture in puree and dry powder of Kakadu plum (Terminalia ferdinandiana) samples using hand-held near infrared spectroscopy. J. Infrared Spectrosc. 29, 201–206. 10.1177/0967033520982361

[B33] Bonapace G. Gentile F. Coppedé N. Coluccio M. L. Garo V. Vismara M. F. M. (2021). Methylglyoxal adducts levels in blood measured on dried spot by portable near-infrared spectroscopy. Nanomaterials 11, 2432. 10.3390/nano11092432 34578748 PMC8472697

[B34] Borba K. R. Aykas D. P. Milani M. I. Colnago L. A. Ferreira M. D. Rodriguez-Saona L. E. (2021). Portable near infrared spectroscopy as a tool for fresh tomato quality control analysis in the field. Appl. Sci. Switz. 11, 3209. 10.3390/app11073209

[B35] Borghi F. T. Santos P. C. Santos F. D. Nascimento M. H. C. Corrêa T. Cesconetto M. (2020). Quantification and classification of vegetable oils in extra virgin olive oil samples using a portable near-infrared spectrometer associated with chemometrics. Microchem. J. 159, 105544. 10.1016/j.microc.2020.105544

[B36] Brasil Y. L. Cruz-Tirado J. P. Barbin D. F. (2022). Fast online estimation of quail eggs freshness using portable NIR spectrometer and machine learning. Food control. 131, 108418. 10.1016/j.foodcont.2021.108418

[B37] Brito A. A. D. Campos F. Nascimento A. D. R. Corrêa G. D. C. Silva F. A. D. Teixeira G. H. D. A. (2021). Determination of soluble solid content in market tomatoes using near-infrared spectroscopy. Food control. 126, 108068. 10.1016/j.foodcont.2021.108068

[B38] Bwambok D. K. Siraj N. Macchi S. Larm N. E. Baker G. A. Pérez R. L. (2020). Qcm sensor arrays, electroanalytical techniques and nir spectroscopy coupled to multivariate analysis for quality assessment of food products, raw materials, ingredients and foodborne pathogen detection: Challenges and breakthroughs. Sens. Switz. 20, 6982–7042. 10.3390/s20236982 PMC7730680 33297345

[B39] Catauro M. Naviglio D. Risoluti R. Vecchio Ciprioti S. (2018). Sol–gel synthesis and thermal behavior of bioactive ferrous citrate–silica hybrid materials. J. Therm. Anal. Calorim. 133, 1085–1092. 10.1007/s10973-018-7137-7

[B40] Caillet C. Vickers S. Zambrzycki S. Fernández F. M. Vidhamaly V. Boutsamay K. (2021). A comparative field evaluation of six medicine quality screening devices in Laos. PLoS Negl. Trop. Dis. 15, e0009674. 10.1371/journal.pntd.0009674 34591852 PMC8483322

[B41] Cavallini N. Pennisi F. Giraudo A. Pezzolato M. Esposito G. Gavoci G. (2022). Chemometric differentiation of sole and plaice fish fillets using three near-infrared instruments. Foods 11, 1643. 10.3390/foods11111643 35681393 PMC9180159

[B42] Cecchini C. Antonucci F. Costa C. Marti A. Menesatti P. (2021). Application of near-infrared handheld spectrometers to predict semolina quality. J. Sci. Food Agric. 101, 151–157. 10.1002/jsfa.10625 32613617

[B43] Chen R. Mei J. Du G. Shi Y. Huang Y. (2022). Convenient detection of white pepper adulteration by portable NIRS and spectral imaging with chemometrics. Microchem. J. 182, 107925. 10.1016/j.microc.2022.107925

[B44] Chen Y. Liu S. Yang Y. Qian Z. Wang B. An C. (2021). On-site determination of heroin by portable near-infrared spectrometer. Aust. J. Forensic Sci. 53, 40–49. 10.1080/00450618.2019.1653370

[B45] Ciaccheri L. Adinolfi B. Mencaglia A. A. Mignani A. G. (2022). Bluetooth-connected pocket spectrometer and chemometrics for olive oil applications. Foods 11, 2265. 10.3390/foods11152265 35954033 PMC9368343

[B46] Clemson B. Mrsan M. Vishwanath K. (2022). “Development of a portable, non-contact diffuse reflectance system for tissue spectroscopy,” in Proc. Prog. Biomed. Opt. Imaging - Proc (USA: SPIE).

[B47] Coombs C. E. O. Liddle R. R. González L. A. (2021). Portable vibrational spectroscopic methods can discriminate between grass-fed and grain-fed beef. J. Infrared Spectrosc. 29, 321–329. 10.1177/09670335211049506

[B48] Coppey F. Bécue A. Sacré P.-Y. Ziemons E. M. Hubert P. Esseiva P. (2020). Providing illicit drugs results in five seconds using ultra-portable NIR technology: An opportunity for forensic laboratories to cope with the trend toward the decentralization of forensic capabilities. Forensic Sci. Int. 317, 110498. 10.1016/j.forsciint.2020.110498 33017781

[B49] Correia R. M. Andrade R. Tosato F. Nascimento M. T. Pereira L. L. Araújo J. B. S. (2020). Analysis of Robusta coffee cultivated in agroforestry systems (AFS) by ESI-FT-ICR MS and portable NIR associated with sensory analysis. J. Food Compos. Anal. 94, 103637. 10.1016/j.jfca.2020.103637

[B50] Crocombe R. A. (2018). Portable spectroscopy. Appl. Spectrosc. 72, 1701–1751. 10.1177/0003702818809719 30335465

[B51] Cruz-Tirado J. P. Lucimar da Silva Medeiros M. Barbin D. F. (2021). On-line monitoring of egg freshness using a portable NIR spectrometer in tandem with machine learning. J. Food Eng. 306, 110643. 10.1016/j.jfoodeng.2021.110643

[B52] Cui J. Cui C. (2021). Non-destructive evaluation of salmon and tuna freshness in a room-temperature incubation environment using a portable visible/near-infrared imaging spectrometer. Trans. ASABE 64, 521–527. 10.13031/trans.13858

[B53] Currò S. Balzan S. Serva L. Boffo L. Ferlito J. C. Novelli E. (2021). Fast and green method to control frauds of geographical origin in traded cuttlefish using a portable infrared reflective instrument. Foods 10, 1678. 10.3390/foods10081678 34441458 PMC8391955

[B54] da Costa Fulgêncio A. C. Resende G. A. P. Teixeira M. C. F. Botelho B. G. Sena M. M. (2022). Determination of alcohol content in beers of different styles based on portable near-infrared spectroscopy and multivariate calibration. Food Anal. Methods 15, 307–316. 10.1007/s12161-021-02126-w

[B55] da Silva Medeiros M. L. Brasil Y. L. Cruz-Tirado L. J. P. Lima A. F. Godoy H. T. Barbin D. F. (2023). Portable NIR spectrometer and chemometric tools for predicting quality attributes and adulteration levels in butteroil. Food control. 144, 109349. 10.1016/j.foodcont.2022.109349

[B56] da Silva Medeiros M. L. Cruz-Tirado J. P. Lima A. F. de Souza Netto J. M. Ribeiro A. P. B. Bassegio D. (2022). Assessment oil composition and species discrimination of brassicas seeds based on hyperspectral imaging and portable near infrared (NIR) spectroscopy tools and chemometrics. J. Food Compos. Anal. 107, 104403. 10.1016/j.jfca.2022.104403

[B57] de Carvalho L. M. Madruga M. S. Estévez M. Badaró A. T. Barbin D. F. (2020). Occurrence of wooden breast and white striping in Brazilian slaughtering plants and use of near-infrared spectroscopy and multivariate analysis to identify affected chicken breasts. J. Food Sci. 85, 3102–3112. 10.1111/1750-3841.15465 32996140

[B58] de Freitas S. T. Guimarães Í. T. Vilvert J. C. do Amaral M. H. P. Brecht J. K. Marques A. T. B. (2022). Mango dry matter content at harvest to achieve high consumer quality of different cultivars in different growing seasons. Postharvest Biol. Technol. 189, 111917. 10.1016/j.postharvbio.2022.111917

[B59] De Jesus Inacio L. Lanza I. Merlanti R. Contiero B. Lucatello L. Serva L. (2020). Discriminant analysis of pyrrolizidine alkaloid contamination in bee pollen based on near-infrared data from lab-stationary and portable spectrometers. Eur. Food Res. Technol. 246, 2471–2483. 10.1007/s00217-020-03590-0

[B60] de Oliveira Aguiar F. C. Dias Guariglia B. A. de Brito A. A. Cardoso Campos L. F. dos Reis Nascimento A. de Carvalho Corrêa G. (2022). Validação prática de modelos de infravermelho próximo para tomate: Sólidos solúveis e acidez. Rev. Cienc. Agrovet. 21, 114–122. 10.5965/223811712122022114

[B61] Deng J. Jiang H. Chen Q. (2022). Characteristic wavelengths optimization improved the predictive performance of near-infrared spectroscopy models for determination of Aflatoxin B1 in maize. J. Cereal Sci. 105, 103474. 10.1016/j.jcs.2022.103474

[B62] Ding L. Gong T. Wang B. Yang Q. Liu W. Pemo R. (2021). Non-invasive study of natural dyes in textiles of the qing dynasty using fiber optic reflectance spectroscopy. J. Cult. Herit. 47, 69–78. 10.1016/j.culher.2020.10.013

[B63] Donis-González I. R. Valero C. Momin M. A. Kaur A. Slaughter D. C. (2020). Performance evaluation of two commercially available portable spectrometers to non-invasively determine table grape and peach quality attributes. Agronomy 10, 148. 10.3390/agronomy10010148

[B64] dos Santos Pereira E. V. de Sousa Fernandes D. D. de Araújo M. C. U. Diniz P. H. G. D. Maciel M. I. S. (2021). *In-situ* authentication of goat milk in terms of its adulteration with cow milk using a low-cost portable NIR spectrophotometer. Microchem. J. 163, 105885. 10.1016/j.microc.2020.105885

[B65] dos Santos V. J. Baqueta M. R. Março P. H. Valderrama P. Visentainer J. V. (2022). Proof-of-Concept on the effect of human milk storage time: Lipid degradation and spectroscopic characterization using portable near-infrared spectrometer and chemometrics. Food Chem. 368, 130675. 10.1016/j.foodchem.2021.130675 34419795

[B66] Duchateau C. Kauffmann J.-M. Canfyn M. Stévigny C. De Braekeleer K. Deconinck E. (2020). Discrimination of legal and illegal cannabis spp. According to European legislation using near infrared spectroscopy and chemometrics. Drug Test. Anal. 12, 1309–1319. 10.1002/dta.2865 32453873

[B67] Eady M. Payne M. Sortijas S. Bethea E. Jenkins D. (2021). A low-cost and portable near-infrared spectrometer using open-source multivariate data analysis software for rapid discriminatory quality assessment of medroxyprogesterone acetate injectables. Spectrochim. Acta - Part Mol. Biomol. Spectrosc. 259, 119917. 10.1016/j.saa.2021.119917 33991812

[B68] Eder G. C. Lin Y. Voronko Y. Spoljaric-Lukacic L. (2020). On-site identification of the material composition of PV modules with mobile spectroscopic devices. Energies 13, 1903. 10.3390/en13081903

[B69] Ehsani S. Dastgerdy E. M. Yazdanpanah H. Parastar H. (2022). Ensemble classification and regression techniques combined with portable near infrared spectroscopy for facile and rapid detection of water adulteration in bovine raw milk. J. Chemom. 37, 3395. 10.1002/cem.3395

[B70] Emsley N. E. M. Holden C. A. Guo S. Bevan R. S. Rees C. McAinsh M. R. (2022). Machine learning approach using a handheld near-infrared (NIR) device to predict the effect of storage conditions on tomato biomarkers. ACS Food Sci. Technol. 2, 187–194. 10.1021/acsfoodscitech.1c00420

[B71] Escuredo O. Meno L. Rodríguez-Flores M. S. Seijo M. C. (2021b). Rapid estimation of potato quality parameters by a portable near-infrared spectroscopy device. Sensors 21, 8222. 10.3390/s21248222 34960316 PMC8707853

[B72] Escuredo O. Rodríguez-Flores M. S. Meno L. Seijo M. C. (2021a). Prediction of physicochemical properties in honeys with portable near-infrared (micronir) spectroscopy combined with multivariate data processing. Foods 10, 317. 10.3390/foods10020317 33546316 PMC7913484

[B73] Esposito G. Sciuto S. Guglielmetti C. Pastorino P. Ingravalle F. Ru G. (2022). Discrimination between wild and farmed sea bass by using new spectrometry and spectroscopy methods. Foods 11, 1673. 10.3390/foods11121673 35741870 PMC9222653

[B74] Fan S. Wang Q. Tian X. Yang G. Xia Y. Li J. (2020). Non-destructive evaluation of soluble solids content of apples using a developed portable vis/NIR device. Biosyst. Eng. 193, 138–148. 10.1016/j.biosystemseng.2020.02.017

[B75] Fazio E. Durso L. Consiglio G. Giuffrida A. Compagnini G. Puglisi O. (2014). Nonlinear scattering and absorption effects in size-selected diphenylpolyynes. J. Phys. Chem. C 118, 28812–28819. 10.1021/jp509666x

[B76] Ferrara G. Marcotuli V. Didonna A. Stellacci A. M. Palasciano M. Mazzeo A. (2022). Ripeness prediction in table grape cultivars by using a portable NIR device. Horticulturae 8, 613. 10.3390/horticulturae8070613

[B77] Fonseca A. C. S. Pereira J. F. Q. Honorato R. S. Bro R. Pimentel M. F. (2022). Hierarchical classification models and handheld NIR spectrometer to human blood stains identification on different floor tiles. Spectrochim. Acta - Part Mol. Biomol. Spectrosc. 267, 120533. 10.1016/j.saa.2021.120533 34749108

[B78] Gaci B. Mas Garcia S. Abdelghafour F. Adrian J. Maupas F. Roger J.-M. (2022). Assessing the potential of a handheld visible-near infrared microspectrometer for sugar beet phenotyping. J. Infrared Spectrosc. 30, 122–129. 10.1177/09670335221083448

[B79] Gales O. Rodemann T. Jones J. Swarts N. (2021). Application of near infra-red spectroscopy as an instantaneous and simultaneous prediction tool for anthocyanins and sugar in whole fresh raspberry. J. Sci. Food Agric. 101, 2449–2454. 10.1002/jsfa.10869 33022086

[B80] Galli A. Gargano M. Bonizzoni L. Bruni S. Interlenghi M. Longoni M. (2021). Imaging and spectroscopic data combined to disclose the painting techniques and materials in the fifteenth century leonardo atelier in milan. Dyes Pigments 187, 109112. 10.1016/j.dyepig.2020.109112

[B81] Galvan D. Lelis C. A. Effting L. Melquiades F. L. Bona E. Conte-Junior C. A. (2022). Low-cost spectroscopic devices with multivariate analysis applied to milk authenticity. Microchem. J. 181, 107746. 10.1016/j.microc.2022.107746

[B82] Gatti R. F. Poppi R. J. Ferreira D. S. (2021). Portable NIR spectrometer for quick identification of fat bloom in chocolates. Food Chem. 342, 128267. 10.1016/j.foodchem.2020.128267 33067047

[B83] Giussani B. Escalante-Quiceno A. T. Boqué R. Riu J. (2021). Measurement strategies for the classification of edible oils using low-cost miniaturised portable nir instruments. Foods 10, 2856. 10.3390/foods10112856 34829136 PMC8618161

[B84] Goi A. Hocquette J.-F. Pellattiero E. De Marchi M. (2022). Handheld near-infrared spectrometer allows on-line prediction of beef quality traits. Meat Sci. 184, 108694. 10.1016/j.meatsci.2021.108694 34700175

[B85] Goisser S. Fernandes M. Wittmann S. Ulrichs C. Mempel H. (2020b). Evaluating the practicability of commercial food-scanners for non-destructive quality assessment of tomato fruit. J. Appl. Bot. Food Qual. 93, 204–214. 10.5073/JABFQ.2020.093.025

[B86] Goisser S. Wittmann S. Fernandes M. Mempel H. Ulrichs C. (2020a). Comparison of colorimeter and different portable food-scanners for non-destructive prediction of lycopene content in tomato fruit. Postharvest Biol. Technol. 167, 111232. 10.1016/j.postharvbio.2020.111232

[B87] Goisser S. Wittmann S. Mempel H. (2021). Food-scanner applications in the fruit and vegetable sector. Landtechnik 76, 52–67. 10.15150/lt.2021.3264

[B88] Gozukara G. Altunbas S. Dengiz O. Adak A. (2022). Assessing the effect of soil to water ratios and sampling strategies on the prediction of EC and PH using PXRF and vis-NIR spectra. Comput. Electron. Agric. 203, 107459. 10.1016/j.compag.2022.107459

[B89] Grassi S. Jolayemi O. S. Giovenzana V. Tugnolo A. Squeo G. Conte P. (2021). Near infrared spectroscopy as a green technology for the quality prediction of intact olives. Foods 10, 1042. 10.3390/foods10051042 34064592 PMC8151771

[B90] Greco V. Giuffrida A. Locatelli M. Savini F. de Grazia U. Ciriolo L. (2023a). Emerging trends in pharmacotoxicological and forensic sample treatment procedures. Appl. Sci. Switz. 13, 2836. 10.3390/app13052836

[B91] Greco V. Locatelli M. Savini F. Grazia U. D. Montanaro O. Rosato E. (2023b). New challenges in (Bio)Analytical sample treatment procedures for clinical applications. Separations 10, 62. 10.3390/separations10010062

[B92] Grossi M. Valli E. Glicerina V. T. Rocculi P. Toschi T. G. Ricco B. (2020). Practical determination of solid fat content in fats and oils by single-wavelength near-infrared analysis. IEEE Trans. Instrum. Meas. 69, 585–592. 10.1109/TIM.2019.2901605

[B93] Guifang W. Hai M. Xin P. (2015). “Identification of varieties of natural textile fiber based on Vis/NIR spectroscopy technology,” in Proceedings of the 2015 IEEE Advanced Information Technology, Electronic and Automation Control Conference (IAEAC), Chongqing, China, 19–20 December 2015 (IEEE), 585–589.

[B94] Gullifa G. Risoluti R. (2021). Innovative miniaturized approach by MicroNIR and chemometrics for the monitoring of the occupational exposure of workers. Proc. J. Phys. Conf. Ser. 1960, 012008. 10.1088/1742-6596/1960/1/012008

[B95] Guo Z. Wang M. Shujat A. Wu J. El-Seedi H. R. Shi J. (2020). Nondestructive monitoring storage quality of apples at different temperatures by near-infrared transmittance spectroscopy. Food Sci. Nutr. 8, 3793–3805. 10.1002/fsn3.1669 32724641 PMC7382128

[B96] Hafiz F. Chalakkal R. J. Hong S. C. Linde G. Hu R. O’Keeffe B. (2022). A new approach to non-mydriatic portable fundus imaging. Expert Rev. Med. Devices 19, 303–314. 10.1080/17434440.2022.2070004 35473498

[B97] Hasan M. K. Aziz M. H. Zarif M. I. I. Hasan M. Hashem M. M. A. Guha S. (2021). Noninvasive hemoglobin level prediction in a mobile phone environment: State of the art review and recommendations. JMIR MHealth UHealth 9, e16806. 10.2196/16806 33830065 PMC8063099

[B98] Hattori Y. Hoshi Y. Hashimoto N. Ichimura Y. Sugiura Y. Otsuka M. (2022). Algorithm and hyperparameter optimizations for hetero-device classification by near-infrared spectra of falsified and substandard amoxicillin capsules. Anal. Sci. 38, 1261–1268. 10.1007/s44211-022-00142-2 35939234

[B99] Hattori Y. Hoshi Y. Ichimura Y. Sugiura Y. Otsuka M. (2021). Device-independent discrimination of falsified amoxicillin capsules using heterogeneous near-infrared spectroscopic devices for training and testing of a support vector machine. Appl. Spectrosc. 75, 1251–1261. 10.1177/0003702821999659 33599512

[B100] He S. Li P. Ren Y. Wei G. Wang Y. Yang Y. (2022). Near-infrared broadband ZnTa2O6:Cr3+Phosphor for pc-LEDs and its application to nondestructive testing. Inorg. Chem. 61, 11284–11292. 10.1021/acs.inorgchem.2c01403 35834349

[B101] Henrique da Silva Melo B. Figueiredo Sales R. da Silva Bastos Filho L. Souza Povoas da Silva J. Gabrielle Carolino de Almeida Sousa A. Maria Camará Peixoto D. (2022). Handheld near infrared spectrometer and machine learning methods applied to the monitoring of multiple process stages in industrial sugar production. Food Chem. 369, 130919. 10.1016/j.foodchem.2021.130919 34461514

[B102] Hespanhol M. C. Souza J. C. Pasquini C. (2020). Feasibility of a portable, low-cost near-infrared spectrophotometer for the quality screening of omega-3 dietary supplements. J. Pharm. Biomed. Anal. 189, 113436. 10.1016/j.jpba.2020.113436 32599486

[B103] Huang W. Luo S. Yang D. Zhang S. (2021). Applications of smartphone-based near-infrared (NIR) imaging, measurement, and spectroscopy technologies to point-of-care (POC) diagnostics [基于智能手机的近红外成像、测量和光谱技术在护理点诊断中的应用]. J. Zhejiang Univ. Sci. B 22, 171–189. 10.1631/jzus.B2000388 33719223 PMC7982329

[B104] Jahangiri B. Barri K. Alavi A. H. Buttlar W. G. (2021). A molecular sensing method integrated with support vector machines to characterize asphalt mixtures. Meas. J. Int. Meas. Confed. 179, 109528. 10.1016/j.measurement.2021.109528

[B105] Jahani R. Yazdanpanah H. van Ruth S. M. Kobarfard F. Alewijn M. Mahboubi A. (2020). Novel application of near-infrared spectroscopy and chemometrics approach for detection of lime juice adulteration. Iran. J. Pharm. Res. 19, 34–44. 10.22037/ijpr.2019.112328.13686 PMC7667562 33224209

[B106] Jiang H. He Y. Chen Q. (2021). Determination of acid value during edible oil storage using a portable NIR spectroscopy system combined with variable selection algorithms based on an MPA-based strategy. J. Sci. Food Agric. 101, 3328–3335. 10.1002/jsfa.10962 33222172

[B107] Jiang H. Liu L. Chen Q. (2022). Rapid determination of acidity index of peanuts by near-infrared spectroscopy technology: Comparing the performance of different near-infrared spectral models. Infrared Phys. Technol. 125, 104308. 10.1016/j.infrared.2022.104308

[B108] Jiang H. Liu T. Chen Q. (2020). Dynamic monitoring of fatty acid value in rice storage based on a portable near-infrared spectroscopy system. Spectrochim. Acta - Part Mol. Biomol. Spectrosc. 240, 118620. 10.1016/j.saa.2020.118620 32599483

[B109] Jianqiang Z. Yan L. Yufeng H. Gangyi H. Nannan B. (2020). Characterization of deep green infection in tobacco leaves using a hand-held digital light projection based near-infrared spectrometer and an extreme learning machine algorithm. Anal. Lett. 53, 2266–2277. 10.1080/00032719.2020.1738452

[B110] Jin G. Wang Y.-J. Li M. Li T. Huang W.-J. Li L. (2021). Rapid and real-time detection of black tea fermentation quality by using an inexpensive data fusion system. Food Chem. 358, 129815. 10.1016/j.foodchem.2021.129815 33915424

[B111] Jorge dos Santos V. Baqueta M. R. Neia V. J. C. Magalhães de Souza P. Março P. H. Valderrama P. (2021). MicroNIR spectroscopy and multivariate calibration in the proximal composition determination of human milk. LWT 147, 111645. 10.1016/j.lwt.2021.111645

[B112] Kappacher C. Trübenbacher B. Losso K. Rainer M. Bonn G. K. Huck C. W. (2022). Portable vs. Benchtop NIR-sensor technology for classification and quality evaluation of black truffle. Molecules 27, 589. 10.3390/molecules27030589 35163862 PMC8838426

[B113] Kaufmann K. C. Sampaio K. A. García-Martín J. F. Barbin D. F. (2022). Identification of coriander oil adulteration using a portable NIR spectrometer. Food control. 132, 108536. 10.1016/j.foodcont.2021.108536

[B114] Khongkaew P. Cruz J. Bertotto J. P. Cárdenas V. Alcalà M. Nuchtavorn N. (2022). A comparative study of benchtop and portable NIR and Raman spectroscopic methods for the quantitative determination of curcuminoids in turmeric powder. Foods 11, 2187. 10.3390/foods11152187 35892772 PMC9331271

[B115] Kovacs Z. Muncan J. Veleva P. Oshima M. Shigeoka S. Tsenkova R. (2022). Aquaphotomics for monitoring of groundwater using short-wavelength near-infrared spectroscopy. Spectrochim. Acta - Part Mol. Biomol. Spectrosc. 279, 121378. 10.1016/j.saa.2022.121378 35617835

[B116] Kranenburg R. F. Ou F. Sevo P. Petruzzella M. de Ridder R. van Klinken A. (2022c). On-site illicit-drug detection with an integrated near-infrared spectral sensor: A proof of concept. Talanta 245, 123441. 10.1016/j.talanta.2022.123441 35405444

[B117] Kranenburg R. F. Ramaker H.-J. Sap S. van Asten A. C. (2022a). A calibration friendly approach to identify drugs of abuse mixtures with a portable near-infrared analyzer. Drug Test. Anal. 14, 1089–1101. 10.1002/dta.3231 35098685 PMC9305489

[B118] Kranenburg R. F. Ramaker H.-J. van Asten A. C. (2022b). On-site forensic analysis of colored seized materials: Detection of Brown heroin and MDMA-tablets by a portable NIR spectrometer. Drug Test. Anal. 14, 1762–1772. 10.1002/dta.3356 35968822 PMC9804980

[B119] Kranenburg R. F. Ramaker H. J. Asten V. Portable A. C. (2022e). Portable near infrared spectroscopy for the isomeric differentiation of new psychoactive substances. Forensic Sci. Int. 341, 111467. 10.1016/j.forsciint.2022.111467 36154979

[B120] Kranenburg R. F. Weesepoel Y. Alewijn M. Sap S. Arisz P. W. F. van Esch A. (2022d). Dataset of near-infrared spectral data of illicit-drugs and forensic casework samples analyzed by five portable spectrometers operating in different wavelength ranges. Data Brief. 45, 108660. 10.1016/j.dib.2022.108660 36425973 PMC9679452

[B121] Kucha C. T. Ngadi M. O. (2020). Rapid assessment of pork freshness using miniaturized NIR spectroscopy. J. Food Meas. Charact. 14, 1105–1115. 10.1007/s11694-019-00360-9

[B122] Kumagai M. Matsuura N. Li H. Ohisa N. Amano T. Ogawa N. (2004). Application of a portable near infrared spectrometer for the manufacturing of noodle products. J. Near Infrared Spectrosc. 12, 127–131. 10.1255/jnirs.417

[B123] Lanza I. Conficoni D. Balzan S. Cullere M. Fasolato L. Serva L. (2021). Assessment of chicken breast shelf life based on bench-top and portable near-infrared spectroscopy tools coupled with chemometrics. Food Qual. Saf. 5, 32. 10.1093/fqsafe/fyaa032

[B124] Li D. Tianyi G. Qin Y. Wei L. Na W. Bo W. (2021b). Non-destructive fiber type identification in ancient textiles using portable near-infrared fiber optic reflectance spectroscopy [便携式近红外光纤光谱在纺织品文物纤维 无损检测中的应用]. Sci. Conserv. Archaeol. 33, 128–138.

[B125] Li Y. Zheng X. Zhang D. Li X. Fang F. Chen L. (2021a). Rapid nondestructive simultaneous detection for physicochemical properties of different types of sheep meat cut using portable vis/NIR reflectance spectroscopy system. Foods 10, 1975. 10.3390/foods10091975 34574084 PMC8468935

[B126] Liang H. Shi Z. Fan Y. Ren Z. Yuan T. Huang Y. (2022). Micro NIR on-site and rapid detection system for cow manure slurry based on cloud sharing of calibration model [基于定标模型云共享的奶牛粪水微型 NIR 现场速测系统]. Nongye Gongcheng XuebaoTransactions Chin. Soc. Agric. Eng. 38, 208–215. 10.11975/j.issn.1002-6819.2022.10.025

[B127] Lim C. H. Sirisomboon P. (2021). Measurement of cross link densities of prevulcanized natural rubber latex and latex products using low-cost near infrared spectrometer. Ind. Crops Prod. 159, 113016. 10.1016/j.indcrop.2020.113016

[B128] Liu L. Yan L. Xie Y. Xu J. (2013). Determination of fiber contents in blended textiles by NIR combined with BP neural network. ISRN Text. 2013, 546481–546485. 10.1155/2013/546481

[B129] Ma T. Inagaki T. Tsuchikawa S. (2021b). Demonstration of the applicability of visible and near-infrared spatially resolved spectroscopy for rapid and nondestructive wood classification. Holzforschung 75, 419–427. 10.1515/hf-2020-0074

[B130] Ma T. Xia Y. Inagaki T. Tsuchikawa S. (2021a). Rapid and nondestructive evaluation of soluble solids content (SSC) and firmness in apple using vis–NIR spatially resolved spectroscopy. Postharvest Biol. Technol. 173, 111417. 10.1016/j.postharvbio.2020.111417

[B131] Malvandi A. Feng H. Kamruzzaman M. (2022a). Application of NIR spectroscopy and multivariate analysis for non-destructive evaluation of apple moisture content during ultrasonic drying. Spectrochim. Acta - Part Mol. Biomol. Spectrosc. 269, 120733. 10.1016/j.saa.2021.120733 34920303

[B132] Malvandi A. Kapoor R. Feng H. Kamruzzaman M. (2022b). Non-destructive measurement and real-time monitoring of apple hardness during ultrasonic contact drying via portable NIR spectroscopy and machine learning. Infrared Phys. Technol. 122, 104077. 10.1016/j.infrared.2022.104077

[B133] Mancini M. Mircoli A. Potena D. Diamantini C. Duca D. Toscano G. (2020). Prediction of pellet quality through machine learning techniques and near-infrared spectroscopy. Comput. Ind. Eng. 147, 106566. 10.1016/j.cie.2020.106566

[B134] Manuel M. N. B. da Silva A. C. Lopes G. S. Ribeiro L. P. D. (2022). One-class classification of special agroforestry Brazilian coffee using NIR spectrometry and chemometric tools. Food Chem. 366, 130480. 10.1016/j.foodchem.2021.130480 34284192

[B135] Manuelian C. L. Ghetti M. De Lorenzi C. Pozza M. Franzoi M. De Marchi M. (2022). Feasibility of pocket-sized near-infrared spectrometer for the prediction of cheese quality traits. J. Food Compos. Anal. 105, 104245. 10.1016/j.jfca.2021.104245

[B136] Maraphum K. Saengprachatanarug K. Wongpichet S. Phuphaphud A. Posom J. (2020). In-field measurement of starch content of cassava tubers using handheld vis-near infrared spectroscopy implemented for breeding programmes. Comput. Electron. Agric. 175, 105607. 10.1016/j.compag.2020.105607

[B137] Maraphum K. Saengprachatanarug K. Wongpichet S. Phuphuphud A. Posom J. (2022). Achieving robustness across different ages and cultivars for an NIRS-PLSR model of fresh cassava root starch and dry matter content. Comput. Electron. Agric. 196, 106872. 10.1016/j.compag.2022.106872

[B138] Materazzi S. Peluso G. Ripani L. Risoluti R. (2017a). High-throughput prediction of AKB48 in emerging illicit products by NIR spectroscopy and chemometrics. Microchem. J. 134, 277–283. 10.1016/j.microc.2017.06.014

[B139] Materazzi S. Risoluti R. Pinci S. Saverio Romolo F. (2017b). New insights in forensic chemistry: NIR/Chemometrics analysis of toners for questioned documents examination. Talanta 174, 673–678. 10.1016/j.talanta.2017.06.044 28738640

[B140] McCormick R. Biegert K. (2021). Non-destructive vis/NIR time-series to model apple fruit maturation on the tree. Acta Hortic. 1311, 131–140. 10.17660/ActaHortic.2021.1311.17

[B141] McVey C. Gordon U. Haughey S. A. Elliott C. T. (2021). Assessment of the analytical performance of three near-infrared spectroscopy instruments (benchtop, handheld and portable) through the investigation of coriander seed authenticity. Foods 10, 956. 10.3390/foods10050956 33925477 PMC8145574

[B142] Melendreras C. Forcada S. Fernández-sánchez M. L. Fernández‐colomer B. Costa‐fernández J. M. López A. (2022). Near-infrared sensors for onsite and noninvasive quantification of macronutrients in breast milk. Sensors 22, 1311. 10.3390/s22041311 35214214 PMC8962988

[B143] Menevseoglu A. (2022). Evaluation of portable vibrational spectroscopy sensors as a tool to detect black cumin oil adulteration. Processes 10, 503. 10.3390/pr10030503

[B144] Menevseoglu A. (2021). Non-destructive detection of sesame oil adulteration by portable FTNIR, FT-MIR, and Raman spectrometers combined with chemometrics. J. Turk. Chem. Soc. Sect. Chem. 8, 775–786. 10.18596/jotcsa.940424

[B145] Mishra P. Marini F. Brouwer B. Roger J. M. Biancolillo A. Woltering E. (2021). Sequential fusion of information from two portable spectrometers for improved prediction of moisture and soluble solids content in pear fruit. Talanta 223, 121733. 10.1016/j.talanta.2020.121733 33298261

[B146] Mishra P. Woltering E. (2021). Handling batch-to-batch variability in portable spectroscopy of fresh fruit with minimal parameter adjustment. Anal. Chim. Acta 1177, 338771. 10.1016/j.aca.2021.338771 34482899

[B147] Mogollón R. Contreras C. da Silva Neta M. L. Marques E. J. N. Zoffoli J. P. de Freitas S. T. (2020). Non-destructive prediction and detection of internal physiological disorders in “keitt” mango using a hand-held vis-NIR spectrometer. Postharvest Biol. Technol. 167, 111251. 10.1016/j.postharvbio.2020.111251

[B148] Moon E. J. Kim Y. Xu Y. Na Y. Giaccia A. J. Lee J. H. (2020). Evaluation of salmon, tuna, and beef freshness using a portable spectrometer. Sens. Switz. 20, 4299–4312. 10.3390/s20154299 PMC7435377 32752216

[B149] Morillas A. V. Frascione N. (2022). Portable near-infrared spectroscopy as a screening test of corrosive solutions concealed in plastic containers. Appl. Sci. Switz. 12, 2770. 10.3390/app12062770

[B150] Mulisa Bobasa E. Dao Thi Phan A. Manolis C. Netzel M. Smyth H. Cozzolino D. (2020). Effect of sample presentation on the near infrared spectra of wild harvest Kakadu plum fruits (Terminalia ferdinandiana). Infrared Phys. Technol. 111, 103560. 10.1016/j.infrared.2020.103560

[B151] Muncan J. Tsenkova R. (2023). Aquaphotomics—exploring water molecular systems in nature. Molecules 28, 2630. 10.3390/molecules28062630 36985601 PMC10059907

[B152] Muñiz R. Cuevas-Valdés M. de la Roza-Delgado B. (2020). Milk quality control requirement evaluation using a handheld near infrared reflectance spectrophotometer and a bespoke mobile application. J. Food Compos. Anal. 86, 103388. 10.1016/j.jfca.2019.103388

[B153] Nagy M. M. Wang S. Farag M. A. (2022). Quality analysis and authentication of nutraceuticals using near ir (NIR) spectroscopy: A comprehensive review of novel trends and applications. Trends Food Sci. Technol. 123, 290–309. 10.1016/j.tifs.2022.03.005

[B154] Naimi S. Ayoubi S. Di Raimo L. A. D. L. Dematte J. A. M. (2022). Quantification of some intrinsic soil properties using proximal sensing in arid lands: Application of vis-NIR, MIR, and PXRF spectroscopy. Geoderma Reg. 28, e00484. 10.1016/j.geodrs.2022.e00484

[B155] Ncama K. Magwaza L. S. Tesfay S. Z. Mditshwa A. Mbili N. C. (2020a). In-field application of portable NIR to assess ‘valencia’ orange fruit maturity. Acta Hortic. 1275, 61–68. 10.17660/ActaHortic.2020.1275.9

[B156] Ncama K. Magwaza L. S. Tesfay S. Z. Mditshwa A. Mbili N. (2020b). *In-situ* assessment of harvest maturity of ‘hass’ avocado (persea americana) using portable vis-NIR spectrometer. Acta Hortic. 1299, 339–346. 10.17660/ActaHortic.2020.1299.51

[B157] Netto J. M. Honorato F. A. Celso P. G. Pimentel M. F. (2023). Authenticity of almond flour using handheld near infrared instruments and one class classifiers. J. Food Compos. Anal. 115, 104981. 10.1016/j.jfca.2022.104981

[B158] Ngo V.-D. Hoang L.-T.-A. Pham V.-C. Ngo V.-H. Tran P.-H. (2022). Estimation of pesticide residues on leafy vegetables using a developed handheld spectrometer. Biointerface Res. Appl. Chem. 12, 8163–8173. 10.33263/BRIAC126.81638173

[B159] Ni D. Smyth H. E. Gidley M. J. Cozzolino D. (2022a). A preliminary study on the utilisation of near infrared spectroscopy to predict age and *in vivo* human metabolism. Spectrochim. Acta - Part Mol. Biomol. Spectrosc. 265, 120312. 10.1016/j.saa.2021.120312 34508927

[B160] Ni D. Smyth H. E. Gidley M. J. Cozzolino D. (2022b). Shedding light on human tissue (*in vivo*) to predict satiation, satiety, and food intake using near infrared reflectance spectroscopy: A preliminary study. Innov. Food Sci. Emerg. Technol. 78, 103033. 10.1016/j.ifset.2022.103033

[B161] Noypitak S. Puttipipatkajorn A. Ruangkhasap S. Terdwongworakul A. Puttipipatkajorn A. (2022). Application of a portable near-infrared spectrometer for rapid, non-destructive evaluation of moisture content in para rubber timber. Wood Sci. Technol. 56, 285–303. 10.1007/s00226-021-01354-x

[B162] Oliveira M. M. Cruz-Tirado J. P. Roque J. V. Teófilo R. F. Barbin D. F. (2020). Portable near-infrared spectroscopy for rapid authentication of adulterated Paprika powder. J. Food Compos. Anal. 87, 103403. 10.1016/j.jfca.2019.103403

[B163] Orsi D. Vaccari M. Baraldi A. Cristofolini L. (2022). A portable NIR fluorimeter directly quantifies singlet oxygen generated by nanostructures for photodynamic therapy. Spectrochim. Acta - Part Mol. Biomol. Spectrosc. 265, 120357. 10.1016/j.saa.2021.120357 34534771

[B164] Ou F. van Klinken A. Ševo P. Petruzzella M. Li C. van Elst D. M. J. (2022). Handheld NIR spectral sensor module based on a fully-integrated detector array. Sensors 22, 7027. 10.3390/s22187027 36146377 PMC9501814

[B165] Ouyang Q. Wang L. Zareef M. Chen Q. Guo Z. Li H. (2020). A feasibility of nondestructive rapid detection of total volatile basic nitrogen content in frozen pork based on portable near-infrared spectroscopy. Microchem. J. 157, 105020. 10.1016/j.microc.2020.105020

[B166] Paiva E. M. Ribessi R. L. Rohwedder J. J. R. (2022). Near-infrared spectra of liquid and gas samples by diffuse reflectance employing benchtop and handheld spectrophotometers. Spectrochim. Acta - Part Mol. Biomol. Spectrosc. 264, 120302. 10.1016/j.saa.2021.120302 34461522

[B167] Panikar S. Li J. Rane V. Gillam S. Callegari G. Kurtyka B. (2021). Integrating sensors for monitoring blend content in a pharmaceutical continuous manufacturing plant. Int. J. Pharm. 606, 120085. 10.1016/j.ijpharm.2020.120085 33737095

[B168] Parastar H. van Kollenburg G. Weesepoel Y. van den Doel A. Buydens L. Jansen J. (2020). Integration of handheld NIR and machine learning to “measure & monitor” chicken meat authenticity. Food control. 112, 107149. 10.1016/j.foodcont.2020.107149 PMC7078282 32195297

[B169] Pasquini C. Hespanhol M. C. Cruz K. A. M. L. Pereira A. F. (2020). Monitoring the quality of ethanol-based hand sanitizers by low-cost near-infrared spectroscopy. Microchem. J. 159, 105421. 10.1016/j.microc.2020.105421 32836390 PMC7430279

[B170] Peng J. Li S. Makar R. S. Li H. Feng C. Luo D. (2022). Proximal soil sensing of low salinity in southern xinjiang, China. Remote Sens. 14, 4448. 10.3390/rs14184448

[B171] Pennisi F. Giraudo A. Cavallini N. Esposito G. Merlo G. Geobaldo F. (2021). Differentiation between fresh and thawed cephalopods using nir spectroscopy and multivariate data analysis. Foods 10, 528. 10.3390/foods10030528 33802548 PMC7999131

[B172] Pham V. Weindorf D. C. Dang T. (2021). Soil profile analysis using interactive visualizations, machine learning, and deep learning. Comput. Electron. Agric. 191, 106539. 10.1016/j.compag.2021.106539

[B173] Pissard A. Marques E. J. N. Dardenne P. Lateur M. Pasquini C. Pimentel M. F. (2021). Evaluation of a handheld ultra-compact NIR spectrometer for rapid and non-destructive determination of apple fruit quality. Postharvest Biol. Technol. 172, 111375. 10.1016/j.postharvbio.2020.111375

[B174] Pomerantsev A. L. Rodionova O. Y. (2021). New trends in qualitative analysis: Performance, optimization, and validation of multi-class and soft models. Trac. Trends Anal. Chem. 143, 116372. 10.1016/j.trac.2021.116372

[B175] Prananto J. A. Minasny B. Weaver T. (2021). Rapid and cost-effective nutrient content analysis of cotton leaves using near-infrared spectroscopy (NIRS). PeerJ 9, e11042. 10.7717/peerj.11042 33763307 PMC7956002

[B176] Pu Y. Pérez-Marín D. O’shea N. Garrido-Varo A. (2021). Recent advances in portable and handheld NIR spectrometers and applications in milk, cheese and dairy powders. Foods 10, 2377. 10.3390/foods10102377 34681426 PMC8535602

[B177] Puttipipatkajorn A. Puttipipatkajorn A. (2020). Development of calibration models for rapid determination of moisture content in rubber sheets using portable near-infrared spectrometers. J. Innov. Opt. Health Sci. 13, 91. 10.1142/S1793545820500091

[B178] Qi Z. Wu X. Yang Y. Wu B. Fu H. (2022). Discrimination of the red Jujube varieties using a portable NIR spectrometer and fuzzy improved linear discriminant analysis. Foods 11, 763. 10.3390/foods11050763 35267396 PMC8909659

[B179] Qiao X. Peng Y. Wang Y. Li L. Zhuang Q. Tian W. (2020). Design of portable device for testing sugar content of apples combined with mobile phones [手机联用的苹果糖度便携式检测装置设计与试验]. Nongye Jixie XuebaoTransactions Chin. Soc. Agric. Mach. 51, 491–498. 10.6041/j.issn.1000-1298.2020.S2.061

[B180] Ramadevi P. Kamalakannan R. Suraj G. P. V Hegde D. Varghese M. (2022). Evaluation of Kraft pulp yield and syringyl/guaiacyl ratio from standing trees (Eucalyptus camaldulensis, E. Urophylla, leucaena leucocephala and Casuarina junghuhniana) using portable near infrared spectroscopy. J. Infrared Spectrosc. 30, 40–47. 10.1177/09670335211063634

[B181] Rego G. Ferrero F. Valledor M. Campo J. C. Forcada S. Royo L. J. (2020). A portable IoT NIR spectroscopic system to analyze the quality of dairy farm forage. Comput. Electron. Agric. 175, 105578. 10.1016/j.compag.2020.105578

[B182] Reinig P. Grüger H. Knobbe J. Pügner T. Meyer S. (2018). “Bringing NIR spectrometers into mobile phones,” in MOEMS and miniaturized systems XVII (Bellingham,WA, USA: SPIE), 97–104.

[B183] Ren G. Zhang X. Wu R. Zhang X. Xie T. Zhang Z. (2022). Digital depiction of the quality of dianhong black tea based on pocket-sized near infrared spectroscopy. Infrared Phys. Technol. 127, 104418. 10.1016/j.infrared.2022.104418

[B184] Ren S. Jia Y. (2023). Near-infrared data classification at phone terminal based on the combination of PCA and CS-rbfsvc algorithms. Spectrochim. Acta - Part Mol. Biomol. Spectrosc. 287, 122080. 10.1016/j.saa.2022.122080 36370633

[B185] Risoluti R. Canepari S. Frati P. Fineschi V. Materazzi S. (2019a). “2^ *n* ^ analytical platform” to update procedures in thanatochemistry: Estimation of post mortem interval in vitreous humor. Anal. Chem. 91, 7025–7031. 10.1021/acs.analchem.9b01443 31059231

[B186] Risoluti R. Caprari P. Gullifa G. Massimi S. Maffei L. Sorrentino F. (2020f). An innovative multilevel test for hemoglobinopathies: TGA/Chemometrics simultaneously identifies and classifies sickle cell disease from thalassemia. Front. Mol. Biosci. 7, 141. 10.3389/fmolb.2020.00141 32850950 PMC7396684

[B187] Risoluti R. Caprari P. Gullifa G. Massimi S. Sorrentino F. Buiarelli F. (2019e). New methods for thalassemia screening: TGA/Chemometrics test is not influenced by the aging of blood samples. Microchem. J. 146, 374–380. 10.1016/j.microc.2019.01.008

[B188] Risoluti R. Caprari P. Gullifa G. Massimi S. Sorrentino F. Maffei L. (2020g). Innovative screening test for the early detection of sickle cell anemia. Talanta 219, 121243. 10.1016/j.talanta.2020.121243 32887134

[B189] Risoluti R. Gregori A. Schiavone S. Materazzi S. (2018b). Click and screen technology for the detection of explosives on human hands by a portable MicroNIR-chemometrics platform. Anal. Chem. 90, 4288–4292. 10.1021/acs.analchem.7b03661 29509004

[B190] Risoluti R. Gullifa G. Battistini A. Materazzi S. (2020b). Development of a “single-click” analytical platform for the detection of cannabinoids in hemp seed oil. RSC Adv. 10, 43394–43399. 10.1039/d0ra07142k 35519692 PMC9058129

[B191] Risoluti R. Gullifa G. Battistini A. Materazzi S. (2019c). Lab-on-Click detection of illicit drugs in oral fluids by MicroNIR-chemometrics. Anal. Chem. 91, 6435–6439. 10.1021/acs.analchem.9b00197 31034204

[B192] Risoluti R. Gullifa G. Battistini A. Materazzi S. (2019d). MicroNIR/chemometrics: A new analytical platform for fast and accurate detection of δ9-tetrahydrocannabinol (THC) in oral fluids. Drug Alcohol Depend. 205, 107578. 10.1016/j.drugalcdep.2019.107578 31610296

[B193] Risoluti R. Gullifa G. Battistini A. Materazzi S. (2020d). Monitoring of cannabinoids in hemp flours by MicroNIR/chemometrics. Talanta 211, 120672. 10.1016/j.talanta.2019.120672 32070595

[B194] Risoluti R. Gullifa G. Battistini A. Materazzi S. (2020c). The detection of cannabinoids in veterinary feeds by MicroNIR/chemometrics: A new analytical platform. Analyst 145, 1777–1782. 10.1039/c9an01854a 31915770

[B195] Risoluti R. Gullifa G. Buiarelli F. Materazzi S. (2020e). Real time detection of amphetamine in oral fluids by MicroNIR/chemometrics. Talanta 208, 120456. 10.1016/j.talanta.2019.120456 31816788

[B196] Risoluti R. Gullifa G. Carcassi E. Masotti A. Materazzi S. (2020a). TGA/Chemometrics addressing innovative preparation strategies for functionalized carbon nanotubes. J. Pharm. Anal. 10, 351–355. 10.1016/j.jpha.2020.02.009 32923009 PMC7474104

[B197] Risoluti R. Gullifa G. Fabiano M. A. Materazzi S. (2015). Biomimetic complexes of Co(II), Mn(II), and Ni(II) with 2-propyl-4,5-imidazoledicarboxylic acid. EGA-MS characterization of the thermally induced decomposition. Russ. J. Gen. Chem. 85, 2374–2377. 10.1134/S1070363215100242

[B198] Risoluti R. Gullifa G. Materazi S. (2020h). Assessing the quality of milk using a multicomponent analytical platform MicroNIR/chemometric. Front. Chem. 8, 614718. 10.3389/fchem.2020.614718 33335892 PMC7736405

[B199] Risoluti R. Materazzi S. Gregori A. Ripani L. (2016). Early detection of emerging street drugs by near infrared spectroscopy and chemometrics. Talanta 153, 407–413. 10.1016/j.talanta.2016.02.044 27130135

[B200] Risoluti R. Materazzi S. (2018). MicroNIR/chemometrics assessement of occupational exposure to hydroxyurea. Front. Chem. 6, 228. 10.3389/fchem.2018.00228 29974049 PMC6020770

[B201] Risoluti R. Materazzi S. Tau F. Russo A. Romolo F. S. (2018a). Towards innovation in paper dating: A MicroNIR analytical platform and chemometrics. Analyst 143, 4394–4399. 10.1039/c8an00871j 30137067

[B202] Risoluti R. Pichini S. Pacifici R. Materazzi S. (2019b). Miniaturized analytical platform for cocaine detection in oral fluids by MicroNIR/chemometrics. Talanta 202, 546–553. 10.1016/j.talanta.2019.04.081 31171220

[B203] Ristova M. Skenderovska M. Jovkovski T. (2022). Nondestructive vis-NIR reflectance spectroscopy as a forensic tool for ink discrimination: A preliminary study. J. Appl. Spectrosc. 89, 967–973. 10.1007/s10812-022-01455-w

[B204] Riu J. Gorla G. Chakif D. Boqué R. Giussani B. (2020). Rapid analysis of milk using low-cost pocket-size NIR spectrometers and multivariate analysis. Foods 9, 1090. 10.3390/foods9081090 32785190 PMC7465951

[B205] Riu J. Vega A. Boqué R. Giussani B. (2022). Exploring the analytical complexities in insect powder analysis using miniaturized NIR spectroscopy. Foods 11, 3524. 10.3390/foods11213524 36360137 PMC9659064

[B206] Rizwana S. Hazarika M. K. (2020). Application of near-infrared spectroscopy for rice characterization using machine learning. J. Inst. Eng. India Ser. A 101, 579–587. 10.1007/s40030-020-00459-z

[B207] Rodionova O. Y. Titova A. V. Pomerantsev A. L. (2016). Discriminant analysis is an inappropriate method of authentication. Trac. Trends Anal. Chem. 78, 17–22. 10.1016/j.trac.2016.01.010

[B208] Rouxinol M. I. Martins M. R. Murta G. C. Barroso J. M. Rato A. E. (2022). Quality assessment of red wine grapes through NIR spectroscopy. Agronomy 12, 637. 10.3390/agronomy12030637

[B209] Rovira G. Miaw C. S. W. Martins M. L. C. Sena M. M. de Souza S. V. C. Callao M. P. (2023). One-class model with two decision thresholds for the rapid detection of cashew nuts adulteration by other nuts. Talanta 253, 123916. 10.1016/j.talanta.2022.123916 36126522

[B210] Rukundo I. R. Danao M.-G. C. MacDonald J. C. Wehling R. L. Weller C. L. (2021b). Performance of two handheld NIR spectrometers to quantify crude protein of composite animal forage and feedstuff. AIMS Agric. Food 6, 462–477. 10.3934/agrfood.2021027

[B211] Rukundo I. R. Danao M.-G. C. Mitchell R. B. Masterson S. D. Weller C. L. (2021a). Comparing the use of handheld and benchtop NIR spectrometers in predicting nutritional value of forage. Appl. Eng. Agric. 37, 171–181. 10.13031/AEA.14157

[B212] Rukundo I. R. Danao M.-G. C. Mitchell R. B. Weller C. L. (2022). Evaluation of predictive performance of PLS regression models after being transferred from benchtop to handheld NIR spectrometers. Biosyst. Eng. 218, 245–255. 10.1016/j.biosystemseng.2022.04.014

[B213] Said M. Wahba A. Khalil D. (2022). Semi-supervised deep learning framework for milk analysis using NIR spectrometers. Chemom. Intell. Lab. Syst. 228, 104619. 10.1016/j.chemolab.2022.104619

[B214] Sandak J. Niemz P. Hänsel A. Mai J. Sandak A. (2021). Feasibility of portable NIR spectrometer for quality assurance in glue-laminated timber production. Constr. Build. Mater. 308, 125026. 10.1016/j.conbuildmat.2021.125026

[B215] Sandak J. Sandak A. Zitek A. Hintestoisser B. Picchi G. (2020). Development of low-cost portable spectrometers for detection of wood defects. Sens. Switz. 20, 545. 10.3390/s20020545 PMC7014491 31963870

[B216] Santonocito R. Tuccitto N. Cantaro V. Carbonaro A. B. Pappalardo A. Greco V. (2022). Smartphone-assisted sensing of trinitrotoluene by optical array. ACS Omega 7, 37122–37132. 10.1021/acsomega.2c02958 36312398 PMC9609071

[B217] Santos C. S. P. Cruz R. Gonçalves D. B. Queirós R. Bloore M. Kovács Z. (2021a). Non-destructive measurement of the internal quality of citrus fruits using a portable NIR device. J. AOAC Int. 104, 61–67. 10.1093/jaoacint/qsaa115 33351939

[B218] Santos F. D. Santos L. P. Cunha P. H. P. Borghi F. T. Romão W. de Castro E. V. R. (2021b). Discrimination of oils and fuels using a portable NIR spectrometer. Fuel 283, 118854. 10.1016/j.fuel.2020.118854

[B219] Santos F. D. Vianna S. G. T. Cunha P. H. P. Folli G. S. de Paulo E. H. Moro M. K. (2022). Characterization of crude oils with a portable NIR spectrometer. Microchem. J. 181, 107696. 10.1016/j.microc.2022.107696

[B220] Santos P. C. Tosato F. Cesconetto M. Corrêa T. Santos F. D. Pires A. A. (2020). Determinação da autenticidade de amostras de azeite comerciais apreendidas no estado do espírito santo usando um espectrofotômetro portátil na região do NIR. Quimica Nova 43, 891–900. 10.21577/0100-4042.20170550

[B221] Sarkar S. Basak J. K. Moon B. E. Kim H. T. (2020). A comparative study of PLSR and SVM-R with various preprocessing techniques for the quantitative determination of soluble solids content of hardy kiwi fruit by a portable vis/NIR spectrometer. Foods 9, 1078. 10.3390/foods9081078 32784804 PMC7466312

[B222] Savoia S. Albera A. Brugiapaglia A. Di Stasio L. Cecchinato A. Bittante G. (2021). Prediction of meat quality traits in the abattoir using portable near-infrared spectrometers: Heritability of predicted traits and genetic correlations with laboratory-measured traits. J. Anim. Sci. Biotechnol. 12, 29. 10.1186/s40104-021-00555-5 33706809 PMC7953783

[B223] Savoia S. Albera A. Brugiapaglia A. Di Stasio L. Ferragina A. Cecchinato A. (2020). Prediction of meat quality traits in the abattoir using portable and hand-held near-infrared spectrometers. Meat Sci. 161, 108017. 10.1016/j.meatsci.2019.108017 31884162

[B224] Scala A. Piperno A. Micale N. Mineo P. G. Abbadessa A. Risoluti R. (2018). Click on PLGA-PEG and hyaluronic acid: Gaining access to anti-leishmanial pentamidine bioconjugates. J. Biomed. Mater. Res. Part B Appl. Biomater. 106, 2778–2785. 10.1002/jbm.b.34058 29219244

[B225] Schoot M. Alewijn M. Weesepoel Y. Mueller-Maatsch J. Kapper C. Postma G. (2022). Predicting the performance of handheld near-infrared photonic sensors from a master benchtop device. Anal. Chim. Acta 1203, 339707. 10.1016/j.aca.2022.339707 35361420

[B226] Shen G. Kang X. Su J. Qiu J. Liu X. Xu J. (2022). Rapid detection of fumonisin B1 and B2 in ground corn samples using smartphone-controlled portable near-infrared spectrometry and chemometrics. Food Chem. 384, 132487. 10.1016/j.foodchem.2022.132487 35189437

[B227] Shrestha G. Calvelo-Pereira R. Roudier P. Martin A. P. Turnbull R. E. Kereszturi G. (2022). Quantification of multiple soil trace elements by combining portable X-ray fluorescence and reflectance spectroscopy. Geoderma 409, 115649. 10.1016/j.geoderma.2021.115649

[B228] Siesler H. W. (2016). “Near infrared spectra, interpretation,” in Encyclopedia of spectroscopy and spectrometry. Editors Lindon J. C. Tranter G. E. Koppenaal D. W. (Oxford, UK: Academic Press), 30–39.

[B229] Silva A. C. D. Ribeiro L. P. D. Vidal R. M. B. Matos W. O. Lopes G. S. (2021). A fast and low-cost approach to quality control of alcohol-based hand sanitizer using a portable near infrared spectrometer and chemometrics. J. Infrared Spectrosc. 29, 119–127. 10.1177/0967033520987315

[B230] Silva L. C. R. Folli G. S. Santos L. P. Barros I. H. A. S. Oliveira B. G. Borghi F. T. (2020). Quantification of beef, pork, and chicken in ground meat using a portable NIR spectrometer. Vib. Spectrosc. 111, 103158. 10.1016/j.vibspec.2020.103158

[B231] Sorak D. Herberholz L. Iwascek S. Altinpinar S. Pfeifer F. Siesler H. W. (2012). New developments and applications of handheld Raman, mid-infrared, and near-infrared spectrometers. Appl. Spectrosc. Rev. 47, 83–115. 10.1080/05704928.2011.625748

[B232] Srinuttrakul W. Mihailova A. Islam M. D. Liebisch B. Maxwell F. Kelly S. D. (2021). Geographical differentiation of hom Mali rice cultivated in different regions of Thailand using ftir-atr and nir spectroscopy. Foods 10, 1951. 10.3390/foods10081951 34441727 PMC8392001

[B233] Sripaurya T. Sengchuai K. Booranawong A. Chetpattananondh K. (2021). Gros Michel banana soluble solids content evaluation and maturity classification using a developed portable 6 channel NIR device measurement. Meas. J. Int. Meas. Confed. 173, 108615. 10.1016/j.measurement.2020.108615

[B234] Sun Y. Wang Y. Huang J. Ren G. Ning J. Deng W. (2020). Quality assessment of instant green tea using portable NIR spectrometer. Spectrochim. Acta - Part Mol. Biomol. Spectrosc. 240, 118576. 10.1016/j.saa.2020.118576 32535491

[B235] Surkova A. Bogomolov A. Paderina A. Khistiaeva V. Boichenko E. Grachova E. (2022). Optical multisensor system based on lanthanide(III) complexes as near-infrared light sources for analysis of milk. Chemosensors 10, 288. 10.3390/chemosensors10070288

[B236] Teixeira A. F. D. S. Andrade R. Mancini M. Silva S. H. G. Weindorf D. C. Chakraborty S. (2022). Proximal sensor data fusion for tropical soil property prediction: Soil fertility properties. J. South Am. Earth Sci. 116, 103873. 10.1016/j.jsames.2022.103873

[B237] Tirkey A. Upadhyay L. S. B. (2021). Microplastics: An overview on separation, identification and characterization of microplastics. Mar. Pollut. Bull. 170, 112604. 10.1016/j.marpolbul.2021.112604 34146857

[B238] Torres I. Sánchez M.-T. Pérez-Marín D. (2020). Integrated soluble solid and nitrate content assessment of spinach plants using portable NIRS sensors along the supply chain. Postharvest Biol. Technol. 168, 111273. 10.1016/j.postharvbio.2020.111273

[B239] Torres I. Sánchez M.-T. Vega-Castellote M. Pérez-Marín D. (2021). Fraud detection in batches of sweet almonds by portable near-infrared spectral devices. Foods 10, 1221. 10.3390/foods10061221 34071284 PMC8229702

[B240] Tosato F. Barros E. V. Cunha D. A. Santos F. D. Corrêa T. Nunes A. (2020). Análise de amOStras de combustíveis por fotometria, NIR portátil E rmn de 1 H – UMA comparação com OS resultadOS encontradOS por técnicas normatizadas. Quimica Nova 43, 155–167. 10.21577/0100-4042.20170473

[B241] Toscano G. Leoni E. Gasperini T. Picchi G. (2022). Performance of a portable NIR spectrometer for the determination of moisture content of industrial wood chips fuel. Fuel 320, 123948. 10.1016/j.fuel.2022.123948

[B242] Trenfield S. J. Tan H. X. Goyanes A. Wilsdon D. Rowland M. Gaisford S. (2020). Non-destructive dose verification of two drugs within 3D printed polyprintlets. Int. J. Pharm. 577, 119066. 10.1016/j.ijpharm.2020.119066 31982555

[B243] Tugnolo A. Giovenzana V. Beghi R. Grassi S. Alamprese C. Casson A. (2021). A diagnostic visible/near infrared tool for a fully automated olive ripeness evaluation in a view of a simplified optical system. Comput. Electron. Agric. 180, 105887. 10.1016/j.compag.2020.105887

[B244] Usman A. G. Ghali U. M. Işik S. (2020). Applications of miniaturized and portable near infrared (NIR), fourier transform infrared (FT-IR) and Raman spectrometers for the inspection and control of pharmaceutical products. Ank. Univ. Eczacilik Fak. Derg. 44, 188–203. 10.33483/jfpau.599077

[B245] van Kollenburg G. Weesepoel Y. Parastar H. van den Doel A. Buydens L. Jansen J. (2020). Dataset of the application of handheld NIR and machine learning for chicken fillet authenticity study. Data Brief. 29, 105357. 10.1016/j.dib.2020.105357 32195297 PMC7078282

[B246] Varrà M. O. Ghidini S. Fabrile M. P. Ianieri A. Zanardi E. (2022). Country of origin label monitoring of musky and common Octopuses (eledone spp. and Octopus vulgaris) by means of a portable near-infrared spectroscopic device. Food control. 138, 109052. 10.1016/j.foodcont.2022.109052

[B247] Vega-Castellote M. Sánchez M.-T. Torres I. de la Haba M.-J. Pérez-Marín D. (2022). Assessment of watermelon maturity using portable new generation NIR spectrophotometers. Sci. Hortic. 304, 111328. 10.1016/j.scienta.2022.111328

[B248] Vera D. A. García H. A. Victoria Waks Serra M. Baez G. R. Iriarte D. I. Pomarico J. A. (2022). A Monte Carlo study of near infrared light propagation in the human head with lesions-a time-resolved approach. Biomed. Phys. Eng. Express 8, 035005. 10.1088/2057-1976/ac59f3 35235912

[B249] Viejo C. G. Caboche C. H. Kerr E. D. Pegg C. L. Schulz B. L. Howell K. (2020). Development of a rapid method to assess beer foamability based on relative protein content using robobeer and machine learning modeling. Beverages 6, 1–11. 10.3390/beverages6020028

[B250] Vinhandelli A. R. De Brito A. A. de Faria R. C. Campos L. F. C. Goulart G. A. S. de Almeida Teixeira G. H. (2023). Near infrared spectroscopy as a tool for agricultural expertise: Identification of tomato seedlings. Acta Sci. - Technol. 45, e61270. 10.4025/actascitechnol.v45i1.61270

[B251] Violino S. Taiti C. Ortenzi L. Marone E. Pallottino F. Costa C. (2022). A ready-to-use portable VIS–NIR spectroscopy device to assess superior EVOO quality. Eur. Food Res. Technol. 248, 1011–1019. 10.1007/s00217-021-03941-5

[B252] Vohland M. Ludwig B. Seidel M. Hutengs C. (2022). Quantification of soil organic carbon at regional scale: Benefits of fusing vis-NIR and MIR diffuse reflectance data are greater for *in situ* than for laboratory-based modelling approaches. Geoderma 405, 115426. 10.1016/j.geoderma.2021.115426

[B253] Wan S.-K. Lü B. Zhang H.-M. He L. Fu J. Ji H.-J. (2021). Quick measurement method of condensation point of diesel based on temperature-compensation model [基于温度修正模型的柴油凝点快速检测方法]. Guang Pu Xue Yu Guang Pu Fen XiSpectroscopy Spectr. Anal. 41, 3111–3116. 10.3964/j.issn.1000-0593(2021)10-3111-06

[B254] Wang F. Zhao C. Xu B. Xu Z. Li Z. Yang H. (2020b). Development of a portable detection device for the quality of fresh tea leaves using spectral technology [便携式茶鲜叶品质光谱检测装置研制]. Nongye Gongcheng XuebaoTransactions Chin. Soc. Agric. Eng. 36, 273–277. 10.3760/cma.j.issn.1673-0860.2020.03.018

[B255] Wang H. Liu X. (2022). Recent developments on broadband near-infrared luminescent materials activated by 3d transition metal ions [3d过渡金属离子激活的宽带近红外发光材料研究进展]. Kuei Suan Jen. Hsueh PaoJournal Chin. Ceram. Soc. 50, 2567–2578. 10.14062/j.issn.0454-5648.22020297

[B256] Wang J. Guo Z. Zou C. Jiang S. El-Seedi H. R. Zou X. (2022a). General model of multi-quality detection for apple from different origins by vis/NIR transmittance spectroscopy. J. Food Meas. Charact. 16, 2582–2595. 10.1007/s11694-022-01375-5

[B257] Wang W. Keller M. D. Baughman T. Wilson B. K. (2020a). Evaluating low-cost optical spectrometers for the detection of simulated substandard and falsified medicines. Appl. Spectrosc. 74, 323–333. 10.1177/0003702819877422 31617368 PMC7066480

[B258] Wang X. Wang L. Fang J. (2021b). Research and application progresses of near-infrared spectral sensing Internet of Things [近红外光谱传感物联网研究与应用进展]. Zhongguo JiguangChinese J. Lasers 48. 10.3788/CJL202148.1210001

[B259] Wang Y.-J. Li T.-H. Li L.-Q. Ning J.-M. Zhang Z.-Z. (2020c). Micro-NIR spectrometer for quality assessment of tea: Comparison of local and global models. Spectrochim. Acta - Part Mol. Biomol. Spectrosc. 237, 118403. 10.1016/j.saa.2020.118403 32361319

[B260] Wang Y. Li M. Li L. Ning J. Zhang Z. (2021c). Green analytical assay for the quality assessment of tea by using pocket-sized NIR spectrometer. Food Chem. 345, 128816. 10.1016/j.foodchem.2020.128816 33316713

[B261] Wang Z. H. Sun P. Y. Wang Y. Z. Wei T. Z. Liu J. (2021a). A measurement method in near infrared spectroscopy for reference correction with the homologous optical beams. J. Instrum. 16, P10019. 10.1088/1748-0221/16/10/P10019

[B262] Wang Z. Yu Y. Wu Y. Gao S. Hu L. Jian C. (2022b). Dynamically monitoring lymphatic and vascular systems in physiological and pathological conditions of a swine model via a portable NIR-II imaging system with ICG. Int. J. Med. Sci. 19, 1864–1874. 10.7150/ijms.71956 36438914 PMC9682514

[B263] Wokadala O. C. Human C. Willemse S. Emmambux N. M. (2020). Rapid non-destructive moisture content monitoring using a handheld portable vis–NIR spectrophotometer during solar drying of mangoes (mangifera indica L.). J. Food Meas. Charact. 14, 790–798. 10.1007/s11694-019-00327-w

[B264] Workman J. Weyer L. (2012). Practical guide and spectral atlas for interpretive near-infrared. Boca Raton, FL, USA: CRC.

[B265] Wu G. Jiang Q. Bai Y. Tian C. Pan W. Jin X. (2020). Nitrogen status assessment for multiple cultivars of strawberries using portable FT-NIR spectrometers combined with cultivar recognition and multivariate analysis. IEEE Access 8, 126039–126050. 10.1109/ACCESS.2020.3007862

[B266] Wu X. Li G. He F. (2021). Nondestructive analysis of internal quality in pears with a self-made near-infrared spectrum detector combined with multivariate data processing. Foods 10, 1315. 10.3390/foods10061315 34200438 PMC8226885

[B267] Xiao P. Chen D. (2022). Near infrared spectra data analysis by using machine learning algorithms. Lect. Notes Netw. Syst. 506, 532–544. 10.1007/978-3-031-10461-9_36

[B268] Xiong Z. Pfeifer F. Siesler H. W. (2016). Evaluating the molecular interaction of organic liquid mixtures using near-infrared spectroscopy. Appl. Spectrosc. 70, 635–644. 10.1177/0003702816631301 26928223

[B269] Yakes B. J. Ellsworth Z. Karunathilaka S. R. Crump E. (2021). Evaluation of portable sensor and spectroscopic devices for seafood decomposition determination. Food Anal. Methods 14, 2346–2356. 10.1007/s12161-021-02064-7

[B270] Yan H. Shen Y. Siesler H. W. (2020). Nah-Infrarot-Spektrometer für Alltagsanwendungen. GIT Labor Fachz 10, 1–4.

[B271] Yan H. Siesler H. W. (2018). Identification of textiles by handheld near infrared spectroscopy: Protecting customers against product counterfeiting. J. Near Infrared Spectrosc. 26, 311–321. 10.1177/0967033518796669

[B272] Yang B. Zhu Z. Gao M. Yan X. Zhu X. Guo W. (2020a). A portable detector on main compositions of raw and homogenized milk. Comput. Electron. Agric. 177, 105668. 10.1016/j.compag.2020.105668

[B273] Yang H. Liu Y. Xiong Z. Liang L. (2020b). Rapid determination of holocellulose and lignin in wood by near infrared spectroscopy and Kernel extreme learning machine. Anal. Lett. 53, 1140–1154. 10.1080/00032719.2019.1700267

[B274] Yang X. Zhu L. Huang X. Zhang Q. Li S. Chen Q. (2022). Determination of the soluble solids content in korla fragrant pears based on visible and near-infrared spectroscopy combined with model analysis and variable selection. Front. Plant Sci. 13, 938162. 10.3389/fpls.2022.938162 35874018 PMC9298609

[B275] Yang Y. Zhang X. Yin J. Yu X. (2020c). Rapid and nondestructive on-site classification method for consumer-grade plastics based on portable NIR spectrometer and machine learning. J. Spectrosc. 2020, 1–8. 10.1155/2020/6631234

[B276] Yao S. Aykas D. P. Rodriguez-Saona L. (2021). Rapid authentication of potato chip oil by vibrational spectroscopy combined with pattern recognition analysis. Foods 10, 42. 10.3390/foods10010042 PMC7824477 33375655

[B277] Yu H.-D. Zuo S.-M. Xia G. Liu X. Yun Y.-H. Zhang C. (2020a). Rapid and nondestructive freshness determination of Tilapia fillets by a portable near-infrared spectrometer combined with chemometrics methods. Food Anal. Methods 13, 1918–1928. 10.1007/s12161-020-01816-1

[B278] Yu H. Liu H. Wang Q. van Ruth S. (2020b). Evaluation of portable and benchtop NIR for classification of high oleic acid peanuts and fatty acid quantitation. LWT 128, 109398. 10.1016/j.lwt.2020.109398

[B279] Yu Y.-Y. Peng Y.-C. Chiu Y.-C. Liu S.-J. Chen C.-P. (2022). Realizing broadband NIR photodetection and ultrahigh responsivity with ternary blend organic photodetector. Nanomaterials 12, 1378. 10.3390/nano12081378 35458086 PMC9027253

[B280] Yu Y. Huang J. Zhu J. Liang S. (2021a). An accurate noninvasive blood glucose measurement system using portable near-infrared spectrometer and transfer learning framework. IEEE Sens. J. 21, 1–3519. 10.1109/JSEN.2020.3025826

[B281] Yu Y. Zhang Q. Huang J. Zhu J. Liu J. (2021b). Nondestructive determination of SSC in korla fragrant pear using a portable near-infrared spectroscopy system. Infrared Phys. Technol. 116, 103785. 10.1016/j.infrared.2021.103785 35471438

[B282] Zambrzycki S. C. Caillet C. Vickers S. Bouza M. Donndelinger D. V. Geben L. C. (2021). Laboratory evaluation of twelve portable devices for medicine quality screening. PLoS Negl. Trop. Dis. 15, e0009360. 10.1371/journal.pntd.0009360 34591844 PMC8483346

[B283] Zhang J. Xu Y. Jiang Y.-W. Zheng C.-Y. Zhou J. Han C.-J. (2021a). Recent advances in application of near-infrared spectroscopy for quality detections of grapes and grape products [近红外光谱技术在葡萄及其制品品质检测中的应用研究进展]. Guang Pu Xue Yu Guang Pu Fen XiSpectroscopy Spectr. Anal. 41, 3653–3659. 10.3964/j.issn.1000-0593(2021)12-3653-07

[B284] Zhang K. Wang H. Zhong L. Liu L. Huang R. Zhang H. (2021b). Evaluation and monitoring of the API content of a portable near infrared instrument combined with chemometrics based on fluidized bed mixing process. J. Pharm. Innov. 17, 1136–1147. 10.1007/s12247-021-09581-2

[B285] Zhang M. Shen M. Pu Y. Li H. Zhang B. Zhang Z. (2022c). Rapid identification of apple maturity based on multispectral sensor combined with spectral shape features. Horticulturae 8, 361. 10.3390/horticulturae8050361

[B286] Zhang T. Wu X. Wu B. Dai C. Fu H. (2022a). Rapid authentication of the geographical origin of milk using portable near-infrared spectrometer and fuzzy uncorrelated discriminant transformation. J. Food Process Eng. 45, 40. 10.1111/jfpe.14040

[B287] Zhang Y. Huang J. Zhang Q. Liu J. Meng Y. Yu Y. (2022b). Nondestructive determination of SSC in an apple by using a portable near-infrared spectroscopy system. Appl. Opt. 61, 3419–3428. 10.1364/AO.455024 35471438

[B288] Zhao Y. Fang S. Ye Y. Yu K. (2021). Chemometric Development Using Portable Molecular Vibrational Spectrometers for Rapid Evaluation of AVC (Valsa Mali Miyabe et Yamada) Infection of Apple Trees. Vib. Spectrosc. 114, 103231. 10.1016/j.vibspec.2021.103231

[B289] Zhong Z. Liu X. Luo X. Zhu Y. Wang S. Huang Y. (2022). Evaluation of coating uniformity for the digestion-aid tablets by portable near-infrared spectroscopy. Int. J. Pharm. 622, 121833. 10.1016/j.ijpharm.2022.121833 35618177

[B290] Zhou L. Tan L. Zhang C. Zhao N. He Y. Qiu Z. (2022b). A portable NIR-system for mixture powdery food analysis using deep learning. LWT 153, 112456. 10.1016/j.lwt.2021.112456

[B291] Zhou L. Wang X. Zhang C. Zhao N. Taha M. F. He Y. (2022a). Powdery food identification using NIR spectroscopy and extensible deep learning model. Food Bioprocess Technol. 15, 2354–2362. 10.1007/s11947-022-02866-5

[B292] Zhu C. Fu X. Zhang J. Qin K. Wu C. (2022a). Review of portable near infrared spectrometers: Current status and new techniques. J. Infrared Spectrosc. 30, 51–66. 10.1177/09670335211030617

[B293] Zhu K. Aykas D. P. Anderson N. Ball C. Plans M. Rodriguez-Saona L. (2022b). Nutritional quality screening of oat groats by vibrational spectroscopy using field-portable instruments. J. Cereal Sci. 107, 103520. 10.1016/j.jcs.2022.103520

[B294] Zhuang X. Su M. Sun Y. Yuan M. Wang L. Zhang Z. (2022). A calibration method based on model updating strategy for the quantitative model of Radix Astragali extract. Microchem. J. 181, 107690. 10.1016/j.microc.2022.107690

